# Genome-wide identification and characterization of cucumber *bHLH* family genes and the functional characterization of *CsbHLH041* in NaCl and ABA tolerance in *Arabidopsis* and cucumber

**DOI:** 10.1186/s12870-020-02440-1

**Published:** 2020-06-11

**Authors:** Jialin Li, Ting Wang, Jing Han, Zhonghai Ren

**Affiliations:** State Key Laboratory of Crop Biology, Shandong Collaborative Innovation Center of Fruit & Vegetable Quality and Efficient Production, Key Laboratory of Biology and Genetic Improvement of Horticultural Crops in Huang-Huai Region, Ministry of Agriculture, College of Horticultural Science and Engineering, Shandong Agricultural University, Tai’an, 271018 Shandong China

**Keywords:** Abiotic stresses, *bHLH* family, Cucumber, *CsbHLH041*, Expression patterns, Regulatory networks

## Abstract

**Background:**

The basic/helix-loop-helix (bHLH) transcription factor family exists in all three eukaryotic kingdoms as important participants in biological growth and development. To date, the comprehensive genomic and functional analyses of *bHLH* genes has not been reported in cucumber (*Cucumis sativus* L.).

**Results:**

Here, a total of 142 *bHLH* genes were identified and classified into 32 subfamilies according to the conserved motifs, phylogenetic analysis and gene structures in cucumber. The sequences of CsbHLH proteins were highly conserved based on the results of multiple sequence alignment analyses. The chromosomal distribution, synteny analysis, and gene duplications of these 142 *CsbHLHs* were further analysed. Many elements related to stress responsiveness and plant hormones were present in the promoter regions of *CsbHLH* genes based on a *cis*-element analysis. By comparing the phylogeny of cucumber and *Arabidopsis* bHLH proteins, we found that cucumber bHLH proteins were clustered into different functional clades of *Arabidopsis* bHLH proteins. The expression analysis of selected *CsbHLHs* under abiotic stresses (NaCl, ABA and low-temperature treatments) identified five *CsbHLH* genes that could simultaneously respond to the three abiotic stresses. Tissue-specific expression profiles of these five genes were also analysed. In addition, *35S*:*CsbHLH041* enhanced the tolerance to salt and ABA in transgenic *Arabidopsis* and in cucumber seedlings, suggesting *CsbHLH041* is an important regulator in response to abiotic stresses. Lastly, the functional interoperability network among the CsbHLH proteins was analysed.

**Conclusion:**

This study provided a good foundation for further research into the functions and regulatory mechanisms of CsbHLH proteins and identified candidate genes for stress resistance in cucumber.

## Background

Basic helix-loop-helix (bHLH) transcription factors form one of the largest families of TFs and exist widely in all three eukaryotic kingdoms [1, 2]. The bHLH TFs are named for their own structural characteristics [3], which are mostly composed of conserved 60 amino acid residues. According to the different functions, they can be divided into two parts: the basic region and the HLH region [4]. The basic region is distributed at the N-terminus of the bHLH conserved domain and contains approximately 15 to 20 residues, which are related to DNA binding [5, 6]. The HLH domain is distributed at the C-terminus of the gene sequence, composing of two amphipathic α-helices mainly constituting of hydrophobic residues linked by a loop region of variable sequence and length. The HLH domain is an essential structure for the formation of homologous or heterologous dimers in bHLH TFs [6, 7].

According to the evolutionary origin, sequence similarity, DNA binding patterns, and functional types, in animals, bHLH transcription factors are mainly divided into six categories, A-F, containing 45 subgroups [8, 9]. In plants, the *bHLH* gene family has been divided into 15–26 groups [10], and even up to 32 groups when atypical bHLH proteins are included [2]. In *Arabidopsis*, 167 bHLH proteins are divided into 21 subfamilies [2, 11]; the 165 bHLH family members in rice are classified into 22 subfamilies [12]; and the 159 bHLH proteins are divided into 21 subfamilies in tomato [13]. Currently, increasing numbers of bHLH proteins have been found in plants, and their functional research is gradually increasing.

In plants, the *bHLH* genes are involved in processes such as metabolic regulation, plant growth and development, and response to environmental signals. The first member of the *bHLH* family discovered was the maize *R* gene, which was shown to play a key role in anthocyanin synthesis [14]. Subsequently, an increasing number of bHLHs have been shown to be involved in a wider range of physiological pathways. For example, Phytochrome Interacting Factors (PIFs) have been reported to respond to light signals [15]; overexpression of *PRE1* activates gibberellin-dependent responses in *Arabidopsis thaliana* [16]; *AtGL3*, *AtEGL3* and *AtTT8* have been demonstrated to be involved in anthocyanin and PA biosynthesis [17, 18]; while *AtGL3*, *AtEGL3* and *AtMYC1* also regulate trichome formation and root hair patterning [19]. In addition, some bHLH TFs are also considered to be able to respond to a variety of abiotic stresses and improve plant stress tolerance, including tolerance to drought tolerance, salt and cold. In wheat, overexpression of *bHLH39* increases tolerance to salt stress [20]. The bHLH TFs often function by forming homodimers or heterodimers with other proteins. For example, MYC3 and MYC4 transcription factors all can interact with multiple JAZ proteins (such as JAZ1, JAZ4, and JAZ9) to jointly regulate the JA signalling pathway [21]. The MYB-bHLH-WD40 complexes are involved in different processes, such as the biosynthesis of anthocyanins and PAs, leaf trichome formation and root hair patterning [22]. In summary, bHLH in plants can form homologous or heterologous complexes with bHLH proteins or other proteins to extend their biological functions.

Cucumber (*Cucumis sativus* L.) is an economically important crop cultivated worldwide [23]. The functions of the *AtbHLH* family have been widely studied in *Arabidopsis thaliana* [2]. However, genome-wide information on members of the *CsbHLH* family has not been reported. In this study, we identified and characterized 142 *bHLH* family genes in cucumber. They were classified into 32 subgroups and could be distributed over seven chromosomes. Their gene structures, conserved motifs, synteny analysis, gene duplications and *cis*-elements in promoters also have been investigated. In addition, the expression levels of some *CsbHLH* genes were measured by qRT-PCR to study their responses to low temperature (4 °C), salt (NaCl) and ABA stress, for which all tested genes were stress-responsive. The protein interaction network among the CsbHLH proteins was predicted, which could help to understand the possible functional mechanism of CsbHLH proteins. Furthermore, overexpression of *CsbHLH041* showed increased salt resistance and ABA resistance compared with controls in cucumber and *Arabidopsis*. We hope that this work will provide useful resources for further studies on the functions and regulatory mechanisms of a potentially important CsbHLH protein, which plays a crucial role in the regulation of abiotic stress responses in cucumber.

## Results

### Identification and analysis of cucumber *bHLH* genes

To identify *CsbHLH* family genes in cucumber, we used the BlastP programme to search against the cucumber genome database by using 166 *Arabidopsis* bHLH proteins [2, 10] and the consensus protein sequences of the bHLH domain, with Hidden Markov Model (HMM) profile (PF00010) as queries. We obtained 164 putative members of the *CsbHLH* family. To confirm the reliability of the *bHLH* genes in the cucumber genome, we used Pfam (http://pfam.janelia.org/) and SMART (http://smart.embl-heidelberg.de/) [24] to search for the presence of the bHLH domain in the amino acid sequences of the 164 proteins. Only 142 proteins had the corresponding conserved bHLH domain, which were named *CsbHLH1* to *CsbHLH142* according to their sequence similarity and phylogenies with individual AtbHLH proteins. Finally, the specific information for the 142 typical bHLH genes, including the gene ID, amino acids length, chromosomal locations, and gene length were present in Table 1. The lengths of the CsbHLH protein sequences varied from 84 residues (*CsaV3_1G005290*) to 960 residues (*CsaV3_1G043790*), and the isoelectric points (pI) varied from 4.57 (*CsaV3_2G030090*) to 11.79 (*CsaV3_6G028530*).

**Table 1:** *bHLH* genes in Cucumber

CsbHLH	Gene ID	Location	Gene length	Amino acid length	pI
*001*	*CsaV3_1G005290*	Chr1:3503806–3,504,411	1909	84	5.04
*002*	*CsaV3_1G011300*	Chr1:6972358–6,976,069	4167	236	5.78
*003*	*CsaV3_3G022420*	Chr3:19737551–19,739,874	958	502	5.7
*004*	*CsaV3_3G049150*	Chr3:40071324–40,074,834	1400	689	5.11
*005*	*CsaV3_3G001710*	Chr3:1295970–1,297,898	5601	643	6.21
*006*	*CsaV3_3G000850*	Chr3:656062–658,628	6057	448	8.65
*007*	*CsaV3_1G039580*	Chr1:24945063–24,953,384	1684	319	5.78
*008*	*CsaV3_2G007370*	Chr2:3725743–3,731,272	605	707	6.09
*009*	*CsaV3_4G032110*	Chr4:22635255–22,641,052	3486	551	6.25
*010*	*CsaV3_1G043790*	Chr1:28804330–28,811,620	6167	960	6.4
*011*	*CsaV3_2G015700*	Chr2:13017831–13,023,291	1802	336	5.64
*012*	*CsaV3_6G000530*	Chr6:351129–356,108	2050	645	5.51
*013*	*CsaV3_3G007980*	Chr3:6919411–6,921,670	2306	650	5.83
*014*	*CsaV3_2G010120*	Chr2:6877325–6,878,870	2524	323	6.02
*015*	*CsaV3_7G025510*	Chr7:14980031–14,984,292	3589	533	6.06
*016*	*CsaV3_6G009090*	Chr6:7311297–7,315,687	3711	486	6.35
*017*	*CsaV3_2G028950*	Chr2:18953613–18,956,324	3528	348	8.3
*018*	*CsaV3_7G007460*	Chr7:4647975–4,650,800	3316	335	6.64
*019*	*CsaV3_6G044570*	Chr6:26373287–26,375,739	3723	330	5.3
*020*	*CsaV3_6G044560*	Chr6:26366139–26,368,813	11,538	309	5.81
*021*	*CsaV3_5G026500*	Chr5:21650877–21,655,135	2678	624	5.04
*022*	*CsaV3_6G044730*	Chr6:26485325–26,487,050	1415	342	4.86
*023*	*CsaV3_2G030090*	Chr2:19685080–19,689,447	4059	363	4.57
*024*	*CsaV3_6G043370*	Chr6:25541506–25,544,586	8321	416	5.2
*025*	*CsaV3_6G044580*	Chr6:26382822–26,384,588	3478	276	6.05
*026*	*CsaV3_2G035250*	Chr2:23586273–23,591,605	3855	239	6.77
*027*	*CsaV3_2G008770*	Chr2:5179234–5,186,154	7290	246	4.92
*028*	*CsaV3_6G008940*	Chr6:7177946–7,179,613	5695	432	5.42
*029*	*CsaV3_6G014370*	Chr6:10430376–10,432,021	6113	308	4.98
*030*	*CsaV3_5G033960*	Chr5:27092009–27,094,880	4184	372	5.77
*031*	*CsaV3_6G036080*	Chr6:20032486–20,036,831	1493	242	9.03
*032*	*CsaV3_1G033410*	Chr1:20481133–20,483,811	5529	256	9.24
*033*	*CsaV3_1G009900*	Chr1:6174783–6,178,372	6920	551	5.6
*034*	*CsaV3_2G001440*	Chr2:370393–376,506	1545	543	8.37
*035*	*CsaV3_1G031920*	Chr1:18957508–18,969,046	2275	242	5.03
*036*	*CsaV3_7G004510*	Chr7:3234760–3,236,331	1320	211	6.77
*037*	*CsaV3_4G034440*	Chr4:24394485–24,395,550	3489	239	7.1
*038*	*CsaV3_4G029740*	Chr4:19342473–19,344,831	5460	253	6.17
*039*	*CsaV3_3G000950*	Chr3:733695–738,681	1011	773	5.11
*040*	*CsaV3_2G026610*	Chr2:18201502–18,202,748	2627	240	7.26
*041*	*CsaV3_1G040580*	Chr1:25826012–25,829,490	1816	492	7.03
*042*	*CsaV3_6G037080*	Chr6:20849605–20,855,801	4137	651	5.91
*043*	*CsaV3_6G041730*	Chr6:24304724–24,306,996	1246	235	6.42
*044*	*CsaV3_1G003910*	Chr1:2423148–2,424,832	2711	266	6.87
*045*	*CsaV3_3G013690*	Chr3:10293079–10,294,740	2245	191	8.64
*046*	*CsaV3_1G006280*	Chr1:4002735–4,008,902	4367	566	9.01
*047*	*CsaV3_1G002260*	Chr1:1450554–1,451,954	1411	261	6.16
*048*	*CsaV3_3G039100*	Chr3:32125102–32,130,846	2115	370	5.74
*049*	*CsaV3_5G033600*	Chr5:26846039–26,850,063	5332	571	6.09
*050*	*CsaV3_1G006650*	Chr1:4277323–4,279,125	2566	279	6.1
*051*	*CsaV3_6G001900*	Chr6:1303975–1,307,218	4986	248	9.26
*052*	*CsaV3_1G037610*	Chr1:23567571–23,568,986	1928	309	4.78
*053*	*CsaV3_6G037070*	Chr6:20836169–20,845,986	5647	263	6.08
*054*	*CsaV3_2G026190*	Chr2:17969523–17,971,339	1844	330	5.15
*055*	*CsaV3_3G034600*	Chr3:29292016–29,296,383	2259	695	5.24
*056*	*CsaV3_3G044120*	Chr3:35995420–35,997,451	4852	272	5.09
*057*	*CsaV3_2G005070*	Chr2:2751170–2,752,663	3663	318	5.74
*058*	*CsaV3_2G014750*	Chr2:12321166–12,324,655	1661	343	6.2
*059*	*CsaV3_7G027630*	Chr7:17195587–17,199,883	1509	317	6
*060*	*CsaV3_2G025890*	Chr2:17778099–17,780,726	3295	342	6.26
*061*	*CsaV3_2G030500*	Chr2:20027274–20,029,389	2181	372	4.71
*062*	*CsaV3_3G015900*	Chr3:11810548–11,813,843	2822	547	6.88
*063*	*CsaV3_7G000080*	Chr7:185656–189,146	2627	457	5.85
*064*	*CsaV3_1G000190*	Chr1:132006–133,915	2323	276	7.03
*065*	*CsaV3_3G028610*	Chr3:25161463–25,169,489	3059	540	5.7
*066*	*CsaV3_2G003660*	Chr2:1833037–1,837,221	6474	422	6.1
*067*	*CsaV3_4G002800*	Chr4:1745825–1,749,669	8026	168	7.65
*068*	*CsaV3_1G045830*	Chr1:31650489–31,656,184	4367	395	6.39
*069*	*CsaV3_4G026430*	Chr4:15704947–15,706,451	3065	196	6.51
*070*	*CsaV3_3G007090*	Chr3:6381666–6,383,510	5744	359	5.94
*071*	*CsaV3_3G049050*	Chr3:40003473–40,010,806	1802	322	7.73
*072*	*CsaV3_1G005810*	Chr1:3727778–3,731,264	2031	443	9.02
*073*	*CsaV3_2G026540*	Chr2:18154564–18,158,701	3855	380	5.01
*074*	*CsaV3_4G035310*	Chr4:24884098–24,888,333	2900	403	5.7
*075*	*CsaV3_3G020750*	Chr3:16961726–16,964,548	7333	248	7.09
*076*	*CsaV3_5G018750*	Chr5:14293739–14,297,926	3510	534	5.2
*077*	*CsaV3_4G034660*	Chr4:24537693–24,539,435	1700	409	6.38
*078*	*CsaV3_7G026520*	Chr7:16047841–16,051,016	3844	490	6.04
*079*	*CsaV3_1G001960*	Chr1:1286661–1,290,828	5186	196	7.64
*080*	*CsaV3_5G040480*	Chr5:31836009–31,840,665	2228	245	5.67
*081*	*CsaV3_6G002130*	Chr6:1472037–1,473,566	1504	161	5.12
*082*	*CsaV3_1G028780*	Chr1:15708212–15,711,935	2358	423	6.34
*083*	*CsaV3_5G024030*	Chr5:18711066–18,713,891	3741	285	6.21
*084*	*CsaV3_4G000380*	Chr4:228607–230,307	5797	342	4.89
*085*	*CsaV3_4G034980*	Chr4:24696914–24,698,535	1065	245	6.18
*086*	*CsaV3_3G014190*	Chr3:10642450–10,643,959	1742	203	7.81
*087*	*CsaV3_7G035000*	Chr7:22142359–22,144,409	1621	394	6.54
*088*	*CsaV3_2G016810*	Chr2:14091802–14,092,813	4235	216	9.77
*089*	*CsaV3_6G012850*	Chr6:8973920–8,976,038	2826	262	9.23
*090*	*CsaV3_5G031540*	Chr5:25725825–25,732,273	1729	679	7.3
*091*	*CsaV3_2G011050*	Chr2:8296805–8,299,080	1004	496	5.5
*092*	*CsaV3_2G013060*	Chr2:10626443–10,627,763	5030	280	9.14
*093*	*CsaV3_3G048260*	Chr3:39394424–39,397,324	4187	326	4.94
*094*	*CsaV3_1G039160*	Chr1:24662874–24,666,933	2825	359	5.94
*095*	*CsaV3_7G008580*	Chr7:5322491–5,324,405	2588	254	7.22
*096*	*CsaV3_2G029940*	Chr2:19575197–19,577,442	4258	308	6.01
*097*	*CsaV3_3G011010*	Chr3:8707121–8,711,973	6448	382	5.08
*098*	*CsaV3_3G021970*	Chr3:19073471–19,076,098	2460	377	5.08
*099*	*CsaV3_6G028530*	Chr6:16812784–16,815,830	2030	207	11.79
*100*	*CsaV3_6G037460*	Chr6:21181685–21,184,516	4024	299	5.66
*101*	*CsaV3_4G029750*	Chr4:19361035–19,364,776	2871	211	9.21
*102*	*CsaV3_6G033930*	Chr6:18737868–18,742,274	1923	333	5.71
*103*	*CsaV3_6G036240*	Chr6:20141242–20,142,683	4656	97	9.18
*104*	*CsaV3_3G012210*	Chr3:9421374–9,425,037	4979	227	5.7
*105*	*CsaV3_3G022870*	Chr3:20405212–20,408,271	3243	236	6.13
*106*	*CsaV3_3G042970*	Chr3:34884792–34,886,594	1529	244	8.4
*107*	*CsaV3_6G018830*	Chr6:13512926–13,515,085	1667	253	7.06
*108*	*CsaV3_2G030310*	Chr2:19895721–19,897,132	4390	240	9.2
*109*	*CsaV3_1G002670*	Chr1:1668618–1,674,219	2118	359	8.65
*110*	*CsaV3_3G045440*	Chr3:37108680–37,112,535	1645	430	6.69
*111*	*CsaV3_5G012430*	Chr5:7900420–7,905,450	2159	451	6.33
*112*	*CsaV3_1G011460*	Chr1:7106592–7,110,120	3046	360	4.75
*113*	*CsaV3_7G008090*	Chr7:5057906–5,059,973	4406	255	6.11
*114*	*CsaV3_5G026380*	Chr5:21538651–21,541,239	4345	173	9.15
*115*	*CsaV3_UNG229040*	scaffold115:93241–95,547	1441	274	9.42
*116*	*CsaV3_3G027730*	Chr3:24067204–24,073,678	9817	529	5.88
*117*	*CsaV3_7G003870*	Chr7:2853486–2,854,424	6196	313	5.32
*118*	*CsaV3_3G016560*	Chr3:12365460–12,367,641	2831	253	8.6
*119*	*CsaV3_1G012350*	Chr1:7668571–7,671,887	2272	205	6.16
*120*	*CsaV3_5G003430*	Chr5:2204438–2,205,442	3080	256	7.75
*121*	*CsaV3_6G047120*	Chr6:27815147–27,818,761	2674	337	6.13
*122*	*CsaV3_1G042640*	Chr1:27559193–27,563,048	2452	438	7.72
*123*	*CsaV3_3G005540*	Chr3:4716554–4,722,201	1766	439	6.41
*124*	*CsaV3_6G046660*	Chr6:27537459–27,541,954	1725	357	8.44
*125*	*CsaV3_5G003420*	Chr5:2191536–2,193,265	1497	261	5.59
*126*	*CsaV3_5G003410*	Chr5:2180337–2,183,163	4495	252	7.01
*127*	*CsaV3_6G049510*	Chr6:28902722–28,905,982	3614	405	5.35
*128*	*CsaV3_4G003860*	Chr4:2368080–2,373,266	3260	357	8.51
*129*	*CsaV3_7G031270*	Chr7:19779388–19,783,470	749	420	8.28
*130*	*CsaV3_3G039080*	Chr3:32107556–32,110,621	3490	367	9.16
*131*	*CsaV3_6G045070*	Chr6:26672336–26,673,833	938	229	10.26
*132*	*CsaV3_6G051560*	Chr6:29996501–29,997,250	1571	250	5.66
*133*	*CsaV3_7G027460*	Chr7:17033739–17,042,484	2825	692	5.66
*134*	*CsaV3_5G037950*	Chr5:30085680–30,087,603	1391	92	9.09
*135*	*CsaV3_1G002240*	Chr1:1440343–1,441,301	2067	93	9.09
*136*	*CsaV3_5G032530*	Chr5:26313766–26,315,796	1914	96	9.17
*137*	*CsaV3_1G009880*	Chr1:6153304–6,155,828	4261	373	6.61
*138*	*CsaV3_7G033460*	Chr7:21082838–21,087,117	3175	298	6.78
*139*	*CsaV3_7G007860*	Chr7:4913547–4,914,938	8745	211	6.35
*140*	*CsaV3_4G010010*	Chr4:7769516–7,771,744	4296	333	5.11
*141*	*CsaV3_1G003270*	Chr1:2026829–2,032,886	4082	619	9.19
*142*	*CsaV3_5G031750*	Chr5:25868912–25,871,372	4279	365	5.84

### Phylogenetic analysis, gene structure and conserved motif analysis of *CsbHLH* gene family

**Fig. 1:**
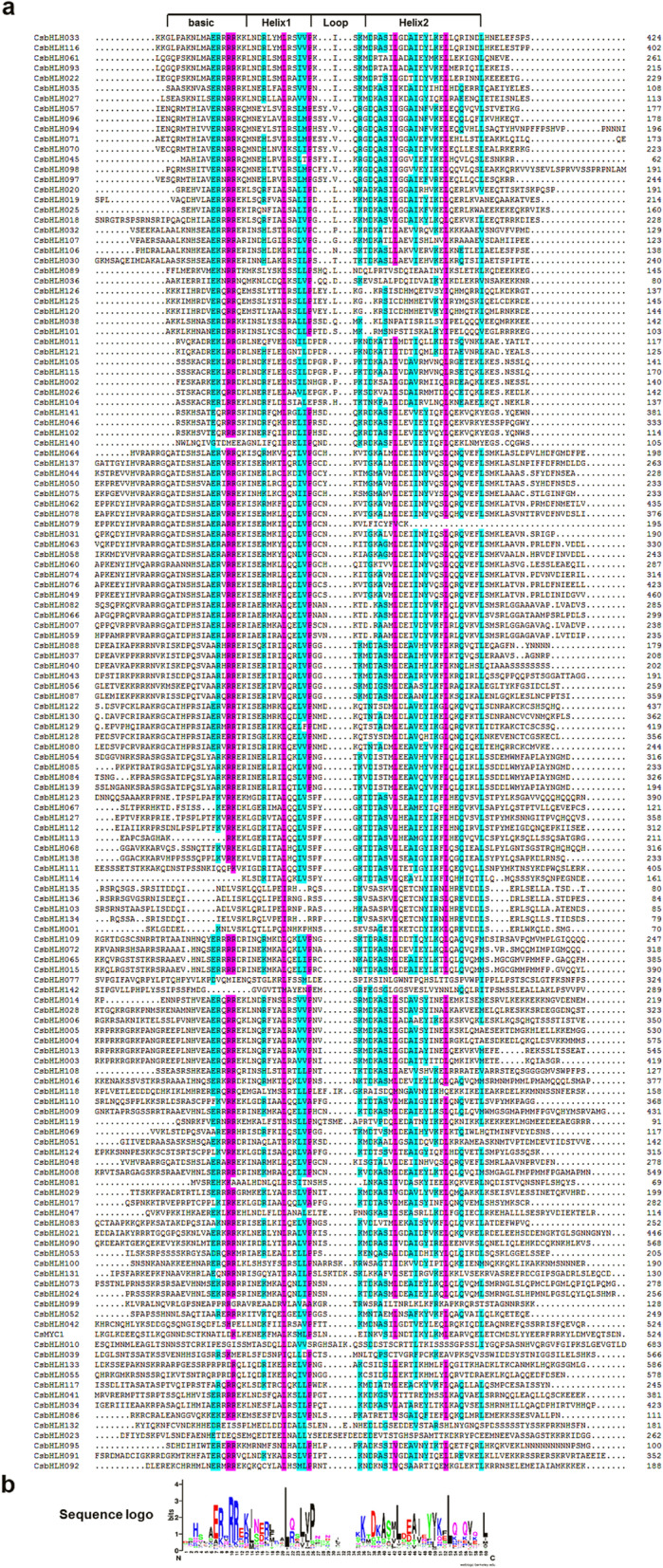
Conserved amino acids and multiple sequence alignment schematic diagrams of the CsbHLHs bHLH domains. (**a**) Multiple sequence alignments of CsbHLH proteins. The CsbHLH conserved sequences were marked with a purple background for an amino acid identity greater than 75% and a light blue background for an amino acid identity greater than 50%. The bHLH domains were labelled. (**b**) Sequence logo of CsbHLH domains. The overall height of each stack represented the conservation of the sequence at that position

**Fig. 2:**
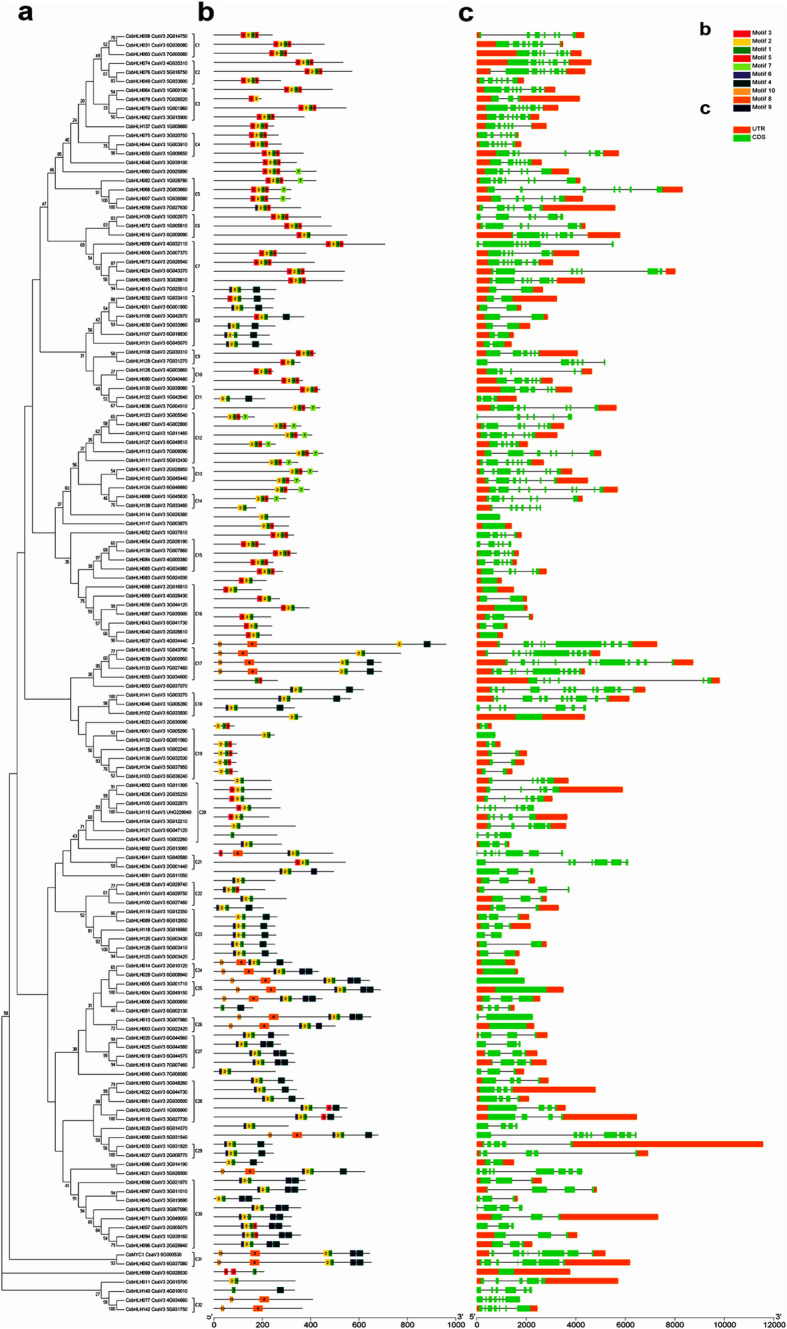
Phylogenetic relationships, gene structure and conserved protein motifs in bHLH genes from cucumber. (**a**) The phylogenetic tree was constructed based on the full-length protein sequences of 142 CsbHLH proteins using MEGA 7.0 software. The tree showed the 32 phylogenetic subgroups (C1-C32) with high bootstrap value. (**b**) Conserved motifs in CsbHLH proteins. The motifs, numbers 1–10, were displayed in different coloured boxes. The sequence logos and *E* values for each motif were shown in Fig. S1. (**c**) Exon-intron structure of *CsbHLH* genes. Exons and introns were indicated by green boxes and single lines, respectively. Blue boxes represented upstream or downstream. The length of each gene was listed in Table 1

To confirm the structural characteristics of CsbHLH proteins, we performed multi-sequence alignment (MSA) analysis on 142 CsbHLH proteins. All 142 CsbHLH proteins contained the characteristic regions of bHLH: two helix regions, one loop region and one basic region (Fig. 1). Additionally, the conserved amino acids with a sequence identity greater than 50% in bHLH domains, were present as light blue or purple colour (Fig. 1a). Sequence logos were produced using the 142 CsbHLH homologous domain amino acid sequences (Fig. 1b). The CsbHLH proteins in cucumber contained 17 conserved amino acids of bHLH domain, which were present in the *bHLH* gene family of *Arabidopsis* and Moso bamboo [2, 25]. As shown in Fig. 1b, we could clearly observe that key amino acid residues Arg-10, Arg-11, Leu-21 and Leu-53 were highly conserved (92, 87, 96, and 90%, respectively) in the 142 CsbHLH proteins. Subsequently, a phylogenetic tree was constructed on the 142 CsbHLH proteins, which were divided into 32 subgroups (C1-C32) based on the clades over 50% bootstrap support (Fig. 2a).

We then performed gene structure analysis of *CsbHLH* gene to support the phylogenetic analysis, which showed that *CsbHLHs* in the same subgroups presented similar numbers of exons and introns, and regardless of intron sizes, the *CsbHLH* genes in the same subgroups had similar intron-exon gene structures (Fig. 2c).

To further investigate the specific motifs of CsbHLH proteins in the same subgroup, we used the MEME tool to identify 10 conserved motifs. The different numbers of conserved motifs were present in 142 CsbHLH proteins (Fig. 2b). Moreover, a similar motif existed in CsbHLH proteins of the same subgroup. For instance, all proteins of subgroup 23 contained motifs 1, 2, 4 and 6, and motif 5 was identified in most CsbHLH proteins. We also found that certain motifs were absent in certain subgroups. For example, motif 4 was absent in all proteins of the 1, 2 and 3 subgroups (Fig. 2b).

In general, the results of conserved motif and gene structure analyses further confirmed the results of the phylogenetic analysis, indicating that proteins within the same subgroup may have similar functions.

### Synteny analysis of *bHLH* genes in cucumber, *Arabidopsis* and tomato

**Fig. 3:**
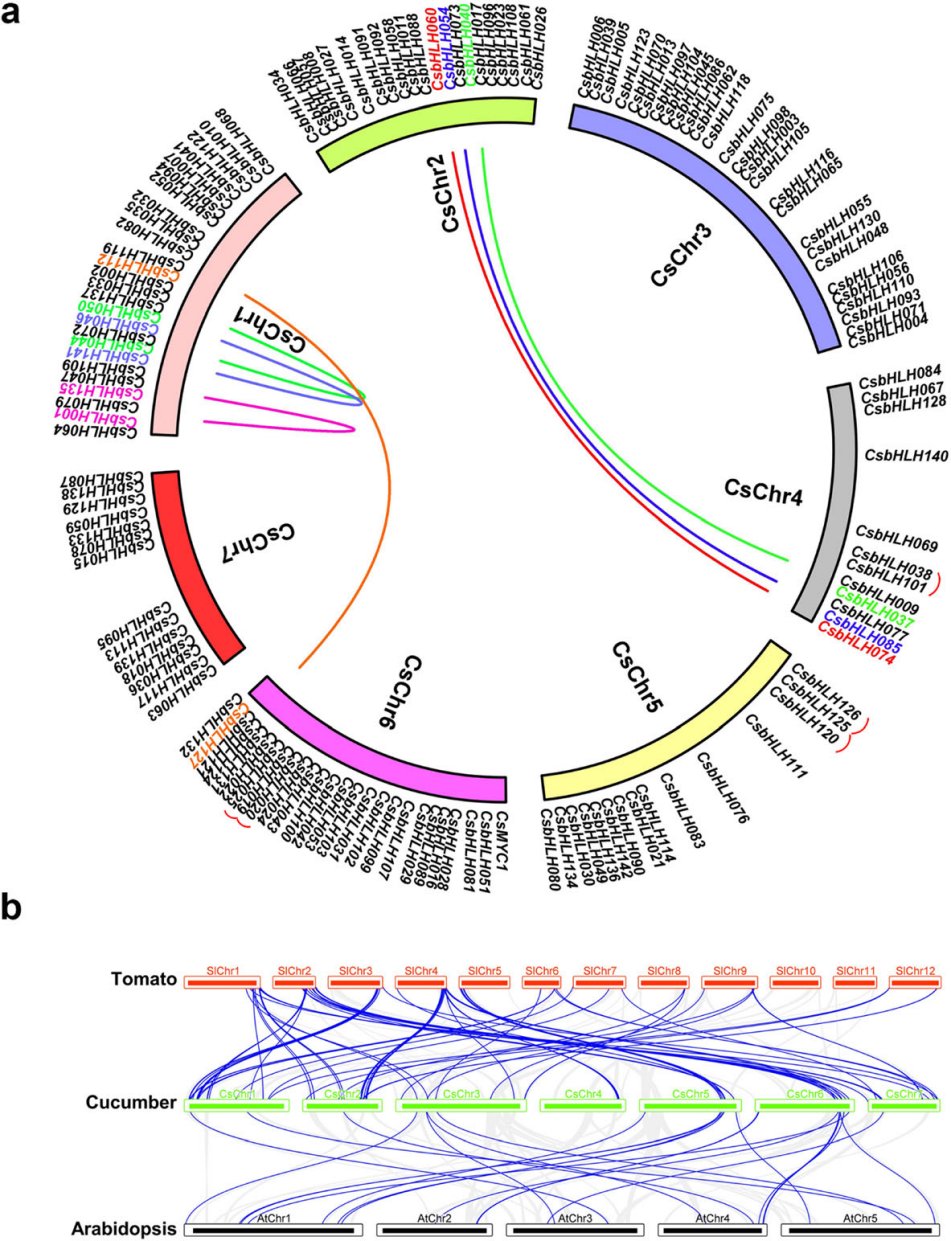
Gene duplication and synteny analysis of *CsbHLH* genes. (**a**) Schematic representations of the chromosomal distribution and interchromosomal relationships of *CsbHLH* genes. Different line colours represented different segmental duplicated *CsbHLH* gene pairs, among which the two genes of the same segmental duplicated gene pair were labelled in the same colour. The red lines in the outer ring indicated tandem duplication gene pairs. (**b**) Synteny analysis of *bHLH* genes between cucumber and *Arabidopsis* and tomato. Blue lines indicated collinear blocks of the *bHLH* gene within the cucumber and *Arabidopsis* and tomato genomes

Through the analysis of the genome distribution of *CsbHLH* genes, we found the 142 *CsbHLH*s (except *CsaV3_UNG229040*) all could be located on chromosomes 1–7 (Fig. 3a; Table 1; Fig. S2). According to the description reported by [26] to determine the duplication of *CsbHLH* genes, we analysed the syntenic regions. The cucumber genome contained 231 segmental duplication blocks and 1468 tandem duplication gene pairs. We obtained five tandem duplication gene pairs (*CsbHLH019* / *CsbHLH020*; *CsbHLH019* / *CsbHLH025*; *CsbHLH120* / *CsbHLH125*; *CsbHLH125* / *CsbHLH126*; *CsbHLH038* / *CsbHLH101*) and seven segmental duplication gene pairs (*CsbHLH112* / *CsbHLH127*; *CsbHLH040* / *CsbHLH037*; *CsbHLH054* / *CsbHLH085*; *CsbHLH060* / *CsbHLH074*; *CsbHLH001* / *CsbHLH135*; *CsbHLH141* / *CsbHLH046*; *CsbHLH050* / *CsbHLH044*) in cucumber *CsbHLH* family (Fig. 3a; Table S1).

In order to further illuminate the phylogenetic mechanisms of *CsbHLH* family, we constructed a comparison of the syntenic map of cucumber related to tomato and *Arabidopsis*, respectively (Fig. 3b). We found that *CsbHLH024*, *CsbHLH040* and *CsbHLH054* genes were associated with more than two syntenic gene pairs between cucumber and tomato. Moreover, for instance, *CsbHLH020* and *CsbHLH049* genes were also corresponded to two syntenic gene pairs between cucumber and *Arabidopsis*, indicating that these *bHLH* genes may play a key role in evolution. In addition, we found certain collinear pairs were present between cucumber and both *Arabidopsis* and tomato (such as *CsbHLH132*, *CsbHLH135* and *CsbHLH136*) (Fig. 3b; Table S2), illustrating that before the ancestral divergence, these orthologous pairs might have already present. Meanwhile, some *CsbHLH* genes were not associated with syntenic gene pairs in *Arabidopsis* or tomato, indicating that they might have been peculiar to cucumber during the course of evolution.

### *Cis*-elements in the promoters of *CsbHLH* genes in cucumber

According to the studies reported by [27], many *bHLH* genes may be able to respond to a variety of abiotic stresses. We isolated the 2-kb promoter regions of the *CsbHLH* genes to identify the potential *cis*-elements (Table S3), in which a number of *CsbHLH* genes particularly presented elements associated with plant hormones (such as auxin, abscisic acid and gibberellic acid) and stress responsiveness (such as drought inducibility and low temperature). Moreover, the promoter regions of some *CsbHLH* genes contained an MYB binding site involved in flavonoid biosynthetic gene regulation, which might be involved in the synthesis of flavonoid in cucumber (Fig. S3; Table S3). In addition, the promoter regions of *CsbHLH* genes contained G-Box and Box-4 elements related to light responsiveness. The *cis*-regulatory elements in *CsbHLH* promoters included the plant light-responsive elements, plant growth- and development-responsive elements, and responding to diverse stresses (Table S3).

To further analyse whether there is co-expression of *CsbHLH* genes with the same *cis*-elements, we constructed a co-expression network of *CsbHLH* genes, based on the available RNA-seq data of 10 cucumber tissues regarding correlations between cucumber *bHLH* genes [26]. The co-expression network containing 23 *CsbHLH* genes (nodes) and 191 correlations (edges) showed that each of the *CsbHLH* genes had multiple co-expression genes with same *cis*-elements (Fig. S4; Table S3). The result indicated the co-expression of genes may be related to the same *cis*-elements in their promoter regions.

### Function prediction of *CsbHLHs* based on phylogenetic analyses

**Fig. 4:**
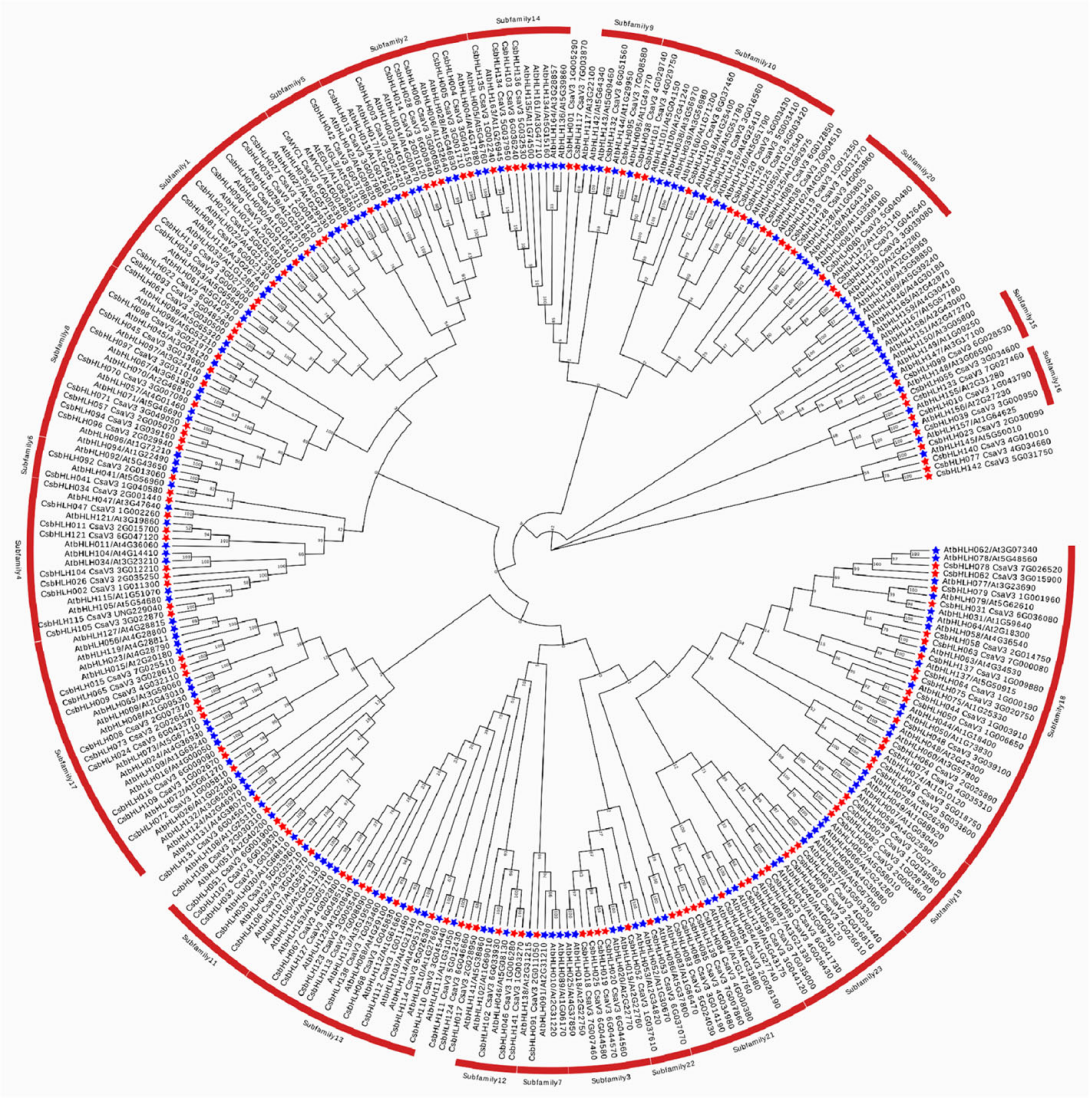
Evolutionary tree analysis (circle tree) and subfamily classifications of bHLHs proteins in cucumber and *Arabidopsis thaliana*. The evolutionary tree was constructed using the Neighbour-Joining method with 1000 bootstrap replication. The evolutionary distances were computed using poisson correction. The analysis involved 142 cucumber bHLH protein sequences and 166 *Arabidopsis thaliana* bHLH protein. Red stars represented the CsbHLH proteins and blue represented the AtbHLH proteins

Previous studies have identified and verified the function of numerous bHLH proteins in *Arabidopsis* [28, 29]. However, the biological functions of CsbHLHs are known little in cucumber. In this study, we performed phylogenetic analyses of 166 AtbHLHs and 142 CsbHLHs proteins to identify the genetic relationship of the bHLH proteins in cucumber and *Arabidopsi*s, so as to preliminarily explore the functions of CsbHLH proteins [2, 10] (Fig. 4).

Finally, we divided the 308 bHLH proteins into 23 subfamilies, and predicted the functions of CsbHLHs according to their verified functional homologs in the same subfamily (Table S4). As shown in Table S4, most of the proteins of subfamilies 1, 2, 4, 10, 13, 14 and 18 responded to different biotic and abiotic stresses [30, 31], such as drought [32], cold [33] and salt [34]. Some of the proteins in subfamilies 4 and 10 might be involved in iron regulation, regulating the iron homeostasis [35]. The proteins of subfamilies 19 and 23 have been identified to regulate flower development [36], and the members of subfamilies 3, 8, 9, 16 and 21 might participate in the development of multiple plant organs [37–39]. There were PIFs in subfamily 17, related to light signal transduction and protect the normal growth and development of plants [15]. The members of subfamily 5 regulate the flavonoid biosynthesis and cell differentiation of root epidermis [22]. The detailed possible functions of CsbHLHs are listed in Table S4.

In general, although the evolutionary relationships could not be clearly deciphered for the functions of all genes, the analysis was meaningful and necessary.

### Expression analysis of *CsbHLH* genes under different stress conditions and in different tissues

**Fig. 5:**
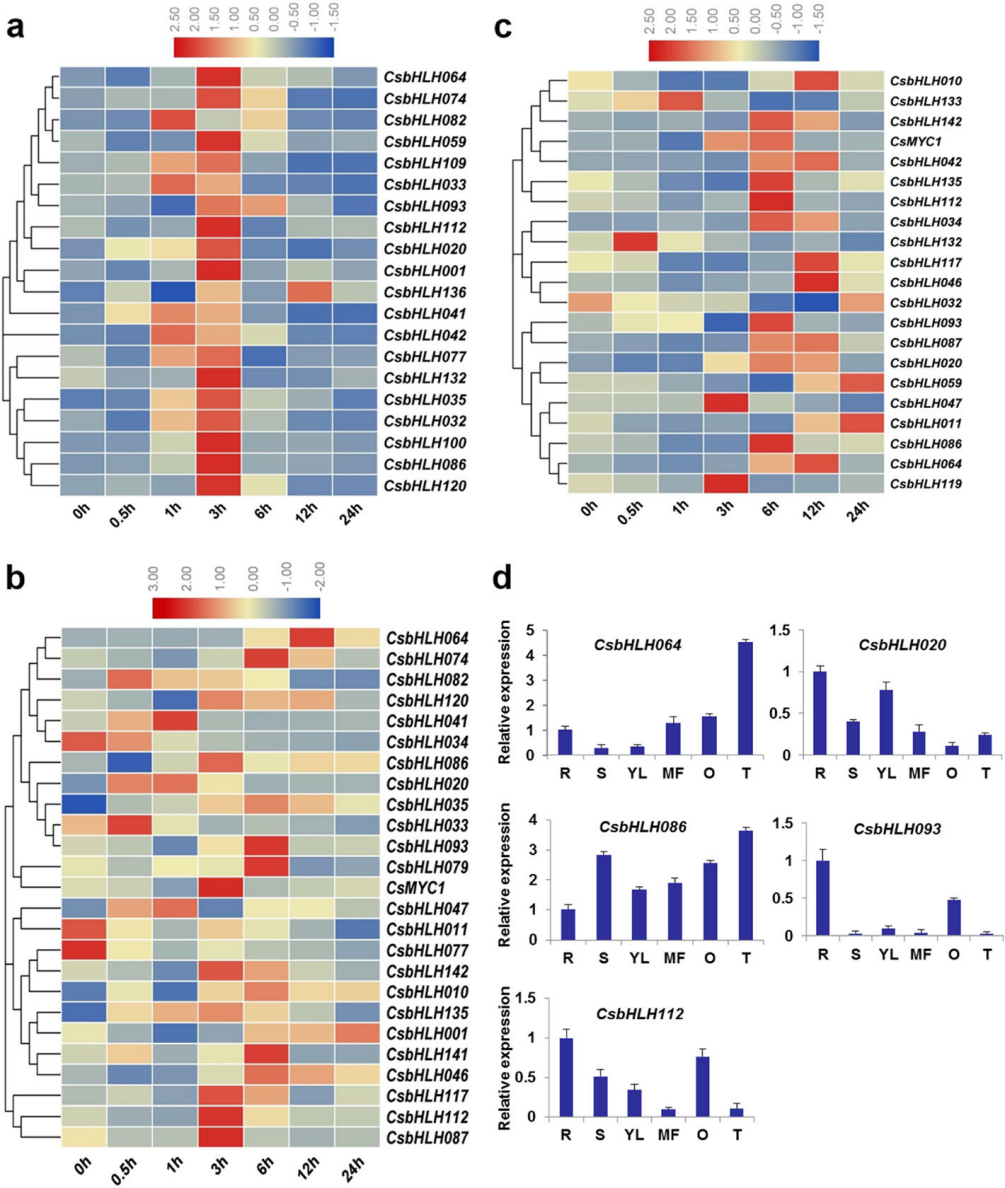
Relative expression analysis of the *CsbHLH* genes under different stress conditions and different tissues. Expression patterns of *CsbHLH* genes under NaCl (100 mM) treatment (**a**), ABA (100 μM) treatment (**b**) and low temperature (4 °C) treatment (**c**). (**d**) Tissue-specific expression profiles of five cucumber *bHLH* genes. Total RNA was isolated from roots (R), stems (S), young leaves (YL), male flowers (MF), ovary (O) and tendrils (T), respectively. The cucumber *β-actin* gene was performed as an internal control, and three independent samples were used for these experiments. Error bars indicated standard errors (SE)

To identify which *CsbHLH* genes play important roles in abiotic stress responses, we carefully screened 21, 20 and 25 bHLH genes based on the *cis*-acting elements containing low temperature, defense and stress responsive and abscisic acid (ABA) elements in the promoters of bHLH genes, respectively, and detected their transcriptional changes with treatments of low temperature (4 °C), salt (NaCl) and ABA, respectively. As expected, all the *CsbHLH* genes screened responded to stress treatments under the respective stress conditions (Fig. 5). For example, the expression levels of the 20 *CsbHLHs* were all positive in response to salt stress, and many of them were upregulated after one hour of salt treatment and achieved the highest expression level 3 h later, and then gradually declined. The expressions of *CsbHLH033*, *CsbHLH041* and *CsbHLH082* were the highest after NaCl treatment for just 1 h, but the expressions levels of *CsbHLH136* reached its maximum after 12 h. *CsbHLH041* was the most susceptible to salt stress (increased by approximately 37-fold) (Fig. 5a). Under ABA treatment, the transcriptional levels of *CsbHLH020*, *CsbHLH041* and *CsbHLH064* were more than 10-fold higher than those of untreated level (*CsbHLH020*: the highest nearly 61-fold; *CsbHLH041*: the highest nearly 55-fold; *CsbHLH064*: the highest nearly 19-fold). In contrast, the expression levels of four of the *CsbHLHs* genes were significantly down-regulated under ABA treatment (*CsbHLH011*, *CsbHLH033*, *CsbHLH034* and *CsbHLH077*), as could be seen in Fig. 5b. The expression levels of 20 of the 21 *CsbHLH* were up-regulated at some time points after the 4 °C treatment, while only *CsbHLH032* was decreased (Fig. 5c). We found the *CsbHLH020*, *CsbHLH064*, *CsbHLH086*, *CsbHLH093* and *CsbHLH112* genes could simultaneously respond to the three abiotic stresses (Fig. 5).

The expression patterns of genes under different conditions are often related to their functions. Therefore, we used qRT-PCR to detect the expression patterns for *CsbHLH020*, *CsbHLH064*, *CsbHLH086*, *CsbHLH093* and *CsbHLH112* abiotic stress-responsive *CsbHLH*s in different tissues. The expression patterns of the five *CsbHLH* genes showed different tissue specificities (Fig. 5d). For instance, *CsbHLH093* and *CsbHLH112* had higher expression levels in ovaries and roots, but lower expression levels in tendrils and male flowers (Fig. 5d). On the contrary, both *CsbHLH064* and *CsbHLH086* were highly expressed in tendrils and male flowers. The expression levels of *CsbHLH020* in young leaves and roots were higher than that in other tissues (Fig. 5d). These results suggested that *CsbHLH* genes might play key roles in plant developmental and physiological processes.

### *CsbHLH041* enhanced tolerance to NaCl and ABA in transgenic *Arabidopsis* and cucumber

**Fig. 6:**
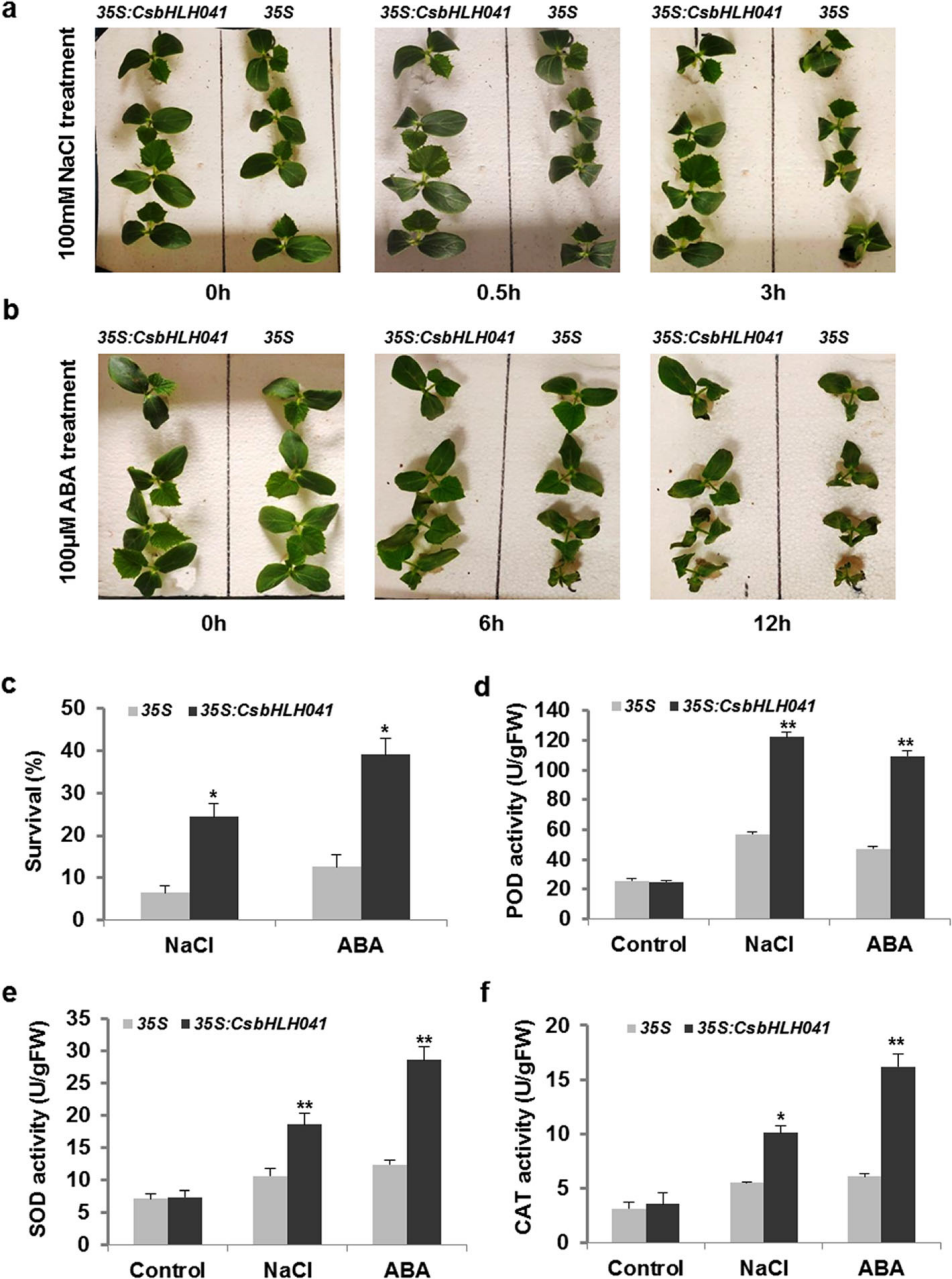
Overexpression of *CsbHLH041* increased salt and ABA tolerance in cucumber seedlings. Phenotypes of *35S* empty vector and *35S*:*CsbHLH041* cucumber seedlings treated with 100 mM NaCl (**a**) and 100 μM ABA (**b**) at different time periods during hydroponic growth. (**c**) Survival of *35S* empty vector and *35S:CsbHLH041* cucumber seedlings after 12 h of salt and ABA treatments. Comparison of the antioxidant enzyme activity between *35S* empty vector and *35S:CsbHLH041* cucumber seedlings under salt and ABA treatment: (**d**) peroxidase (POD) activity, (**e**) superoxide dismutase (SOD) activity, (**f**) catalase (CAT) activity. The bars showed the SE. * and ** indicate significant differences at *P* < 0.05 and *P* < 0.01, respectively

*CsbHLH041* expression was significantly induced by salt and ABA in cucumber (Fig. 5a-b). Therefore, we used *Agrobacterium*-mediated transient transformation of cucumber cotyledons to clarify *CsbHLH041* tolerance to salt and ABA. After 0.5 h of 100 mM NaCl treatment, serious wilting occurred in the seedlings overexpressing *35S* empty vector compared with over-expression *CsbHLH041*, and the wilting difference was more obvious after 3 h of NaCl treatment (Fig. 6a). After 12 h, the survival rate of the transgenic seedlings (24%) was markedly higher than that of the *35S* empty vector seedlings (6%), showing that over-expression of *CsbHLH041* resulted in significant salt resistance (Fig. 6c). After 6 h of ABA treatment, the transgenic seedlings were more vigorous than *35S* empty vector seedlings (Fig. 6b). With the extension of ABA treatment time, the *35S* cucumber seedlings showed visible symptoms of ABA-induced damage, such as drying, wilting, and even death, with survival of only 12%. While some *CsbHLH041* transgenic plants remained green with expanded cotyledons, and the survival rate was up to approximately 40% (Fig. 6b-c).

To clarify the possible factors underlying the enhanced NaCl and ABA resistance, we examined the enzymatic activities in the ROS clearance system under NaCl and ABA treatments, respectively. Without the NaCl or ABA treatment, the enzymatic activities of POD, SOD and CAT in *35S* and *35S*:*CsbHLH041* transgenic seedlings were no significant difference (Fig. 6d-f). Nevertheless, both NaCl treatment and ABA treatment could significantly activate more enzymatic scavenging activities in the *CsbHLH041* transgenic plants than in the *35S* empty vector seedlings (Fig. 6d-f).

**Fig. 7:**
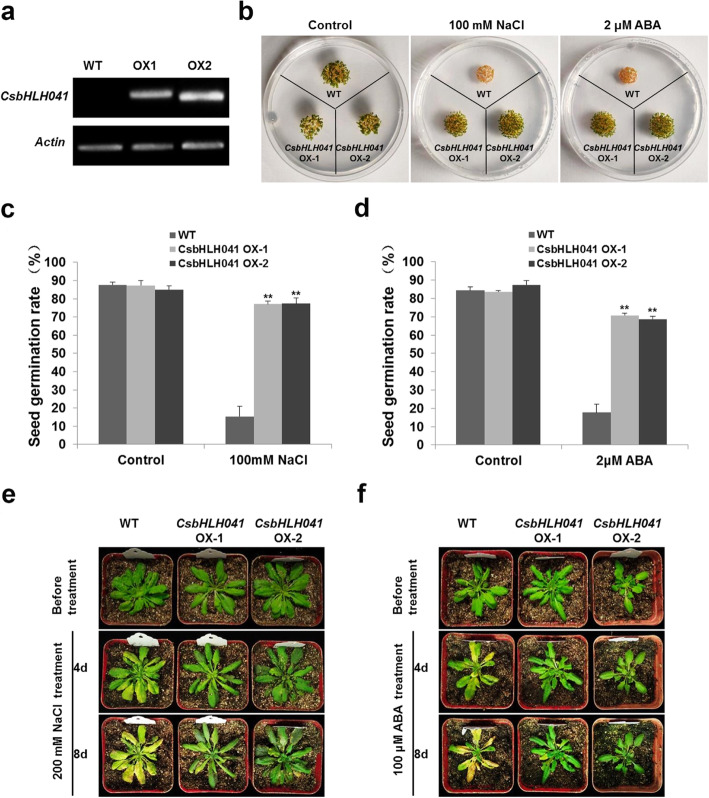
*CsbHLH041* transgenic *Arabidopsis* showed enhanced salt and ABA tolerance. (**a**) Relative expression of *CsbHLH041* in Col-0 (WT) and two T3 generation transgenic lines by semi-quantitative PCR. The *actin8* gene was used as an internal control. The original, uncropped gel image was provided as Additional file 9. (**b**) Germination of WT seeds of Col-0 and *CsbHLH041* transgenic lines OX-1, OX-2 on 1/2 MS supplemented with 100 mM NaCl and 2 μM ABA after 7 days of cultivation at 22 °C. (**c**) and (**d**) Seed germination rate for the corresponding (**b**), respectively. Three biological replications were performed. Asterisks indicated a significant difference **p < 0.01 compared with the corresponding controls. The growth of Col-0 (WT) and *CsbHLH041* transgenic lines after 200 mM NaCl (**e**) and 100 μM ABA (**f**) treatments

To further explore the function of *CsbHLH041* resistance to abiotic stress in plants, transgenic *Arabidopsis* plants overexpressing *CsbHLH041* driven by the *CaMV35S* promoter were generated. Two independent homozygous lines with relatively high expression levels, *CsbHLH041* OX1 and *CsbHLH041* OX2, were selected for the analysis (Fig. 7a). The salt and ABA tolerance of *CsbHLH041* transgenic plants were assessed. There were no differences in seed germination between WT and *CsbHLH041* transgenic *Arabidopsis* on 1/2 MS (Control) (Fig. 7b). However, the germination ratio of transgenic plants seeds was markedly higher than WT seeds in 1/2 MS medium containing 100 mM NaCl or 2 μM ABA (Fig. 7b-d). Subsequently, the 3-week-old seedlings of *CsbHLH041* transgenic lines and wild-type (WT) plants were treated with 200 mM NaCl and 100 μM ABA, respectively. The leaves of WT plants turned severely yellow after 4 days of 200 mM NaCl or 100 μM ABA treatment, while *CsbHLH041* transgenic lines were still growing with green leaves (Fig. 7e-f). After 8 days, the difference in NaCl or ABA resistance between WT plants and *CsbHLH041* transgenic lines was more obvious, which suggested that *CsbHLH041* transgenic plants were more tolerant to salt and ABA stresses than WT.

### The protein interaction network predictions for CsbHLH orthologs in *Arabidopsis* that were crucial for the abiotic stress response

**Fig. 8:**
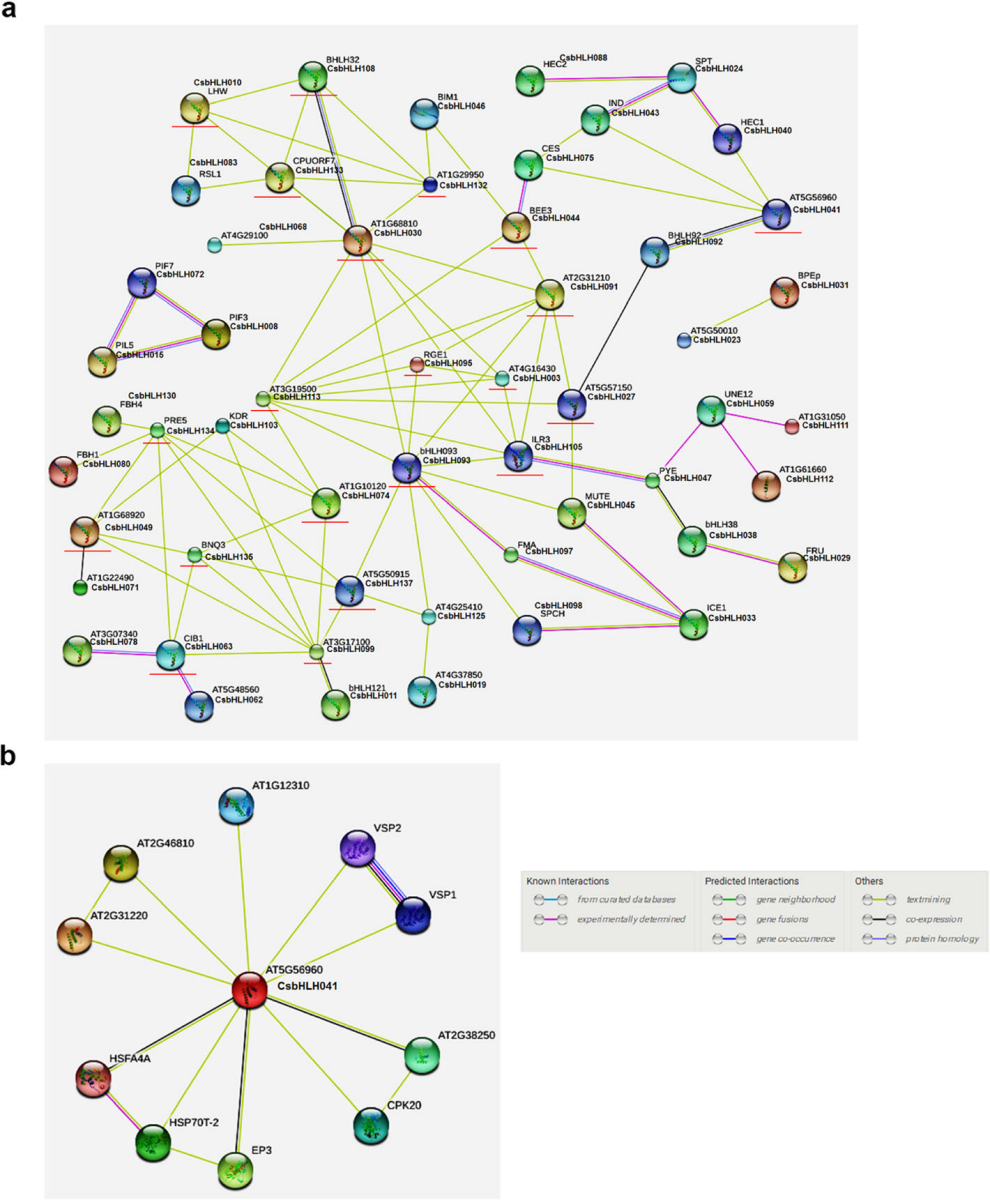
Protein interaction network for CsbHLHs based on CsbHLH orthologs in *Arabidopsis*. Protein interaction network predictions of CsbHLHs (**a**) and CsbHLH041 (**b**). Red lines indicated proteins that were predicted to interact with more than four other bHLH proteins. CsbHLH proteins were shown next to *Arabidopsis* orthologs

Network interaction analysis has been demonstrated to be an effective method to analyse the gene function [40]. We used the software STRING 10 to predict the protein interaction network among the 142 CsbHLH proteins (Fig. 8a). Numerous CsbHLH transcription factors interacted with multiple CsbHLHs, consistent with previous reports demonstrating that the binding activity of specific DNA sequences depends on the homodimers or heterodimers formed by the interactions of bHLH proteins [2]. As shown in Fig. 8a, there were 21 proteins that had correlation with more than four other bHLH proteins, which may make them play important roles in regulating plant stress responses and growth, and detailed informations about these orthologs were showed in Table S6.

In our study, *CsbHLH041* responded significantly to salt and ABA treatments, and *CsbHLH041* could enhance tolerance to NaCl and ABA in transgenic *Arabidopsis* and cucumber (Fig. 5a-b; Fig. 6; Fig. 7). The function of bHLH proteins are mainly realized through the formation of heterodimers or homodimers with other transcription factors, which are essential for their binding to downstream target genes [2]. *AT5G56960*, the *CsbHLH041* homologous gene, was at the centre of the protein association network, indicating that it played main roles in regulating different functional proteins (Fig. 8b; Table S6). For example, EP3 might play a role in both normal plant growth and disease resistance [41]. VSP1 and VSP2 are anti-insect proteins and respond to methyl jasmonate and wounding, in which their defense function were correlated with its acid phosphatase activity [42, 43]. The predicted gene association network provides useful resources for subsequent research.

## Discussion

### Characterization of the cucumber *bHLH* family

The basic helix-loop-helix (bHLH) transcription factor family is the second largest family in eukaryotes [10, 44] and extensive studies of *bHLH* families have been identified in various plants [2]. For example, 166 *bHLH* genes have been identified in *Arabidopsis* [2, 10], 115 *bHLH* genes in *Nelumbo nucifera* [45], 188 *bHLH* genes in apple [40], 167 *bHLH* genes in rice [12] and 159 *bHLH* genes in tomato [13]. The bHLH TFs have been involved in multiple biological processes in plants, especially in regulating defense against biotic and abiotic stresses [46]. However, we know very little about *bHLHs* in cucumber. In our study, 142 *bHLH* cucumber genes were identified and characterized. According to phylogenetic analyses, the 142 CsbHLHs were divided into 32 subgroups (Fig. 2a), and multiple sequence analysis indicated that the conserved bHLH domains existed in all 142 CsbHLH proteins (Fig. 1). For instance, the two amino acid residues Leu-21 and Leu-53 were relatively conserved in the helical region that are essential for the formation of dimers. Moreover, the conservative sequence analyses indicated that almost all 142 CsbHLH proteins had the conserved 1 and 2 motifs. The analyses of gene structure and the motif further supported the phylogenetic relationship for the 142 *CsbHLH* genes (Fig. 2b-c). To sum up, these results showed that all 142 *CsbHLHs* had the characteristics of the *bHLH* family, confirming the reliability of the *bHLH* genes discovered in cucumber.

### Phylogenetic analysis and evolution of cucumber *bHLH* genes

In the model plant *Arabidopsis*, the bHLH gene family has been systematically analysed [2, 11]. To explore the evolutionary relationships between 142 CsbHLH proteins in cucumber and 166 AtbHLH proteins in *Arabidopsis*, a phylogenetic tree was constructed based on the protein of 308 bHLHs, which clustered into 23 subfamilies (Fig. 4). There are differences in anatomy and physiology between cucumber and *Arabidopsis*, so some clades may have different modes of expansion in the *bHLH* family of cucumber and *Arabidopsis*. As shown in Fig. 4 and Table S4, not all bHLH members in cucumber were included in these 23 subfamilies, which suggested that there were differences between cucumber and *Arabidopsis* during the process of evolution.

Studies had shown that gene duplication events played a crucial role in the rapid expansion and evolution of gene families [26]. In the cucumber genome, we identified 231 segmental duplication events and 1468 tandem duplication gene pairs (Table S1). Seven segmental duplication events and five tandem duplication gene pairs were found in the *CsbHLH* family (Fig. 3a). In general, the gene functions of a clade are highly conserved among different plant species, but it is not absolute. Therefore, it is of great significance to accurately identify the true orthologs between plant species based on synteny analysis. The results showed that the cucumber genome had extensive synteny with the *Arabidopsis* and tomato genomes, and 944 and 983 syntenic blocks between the cucumber and *Arabidopsis* and tomato genome were identified, respectively (Table S5). Many *CsbHLH* genes showed a linear relationship with the tomato and *Arabidopsis* genes, respectively (Fig. 3b; Table S2).

Previous studies have shown that orthologous genes are usually distributed in the same clade, and have similar functions. In our study, many CsbHLH proteins were grouped into some functional clades of *Arabidopsis*, providing valuable information for studying the functions of *CsbHLHs*. CsMYC1 and CsbHLH042 were grouped into subfamily5 along with AtGL3, AtEGL3, AtMYC1 and AtTT8, and were highly homologous to these proteins. In *Arabidopsis*, AtGL3, AtEGL3 and AtTT8 have been demonstrated to be key regulators of anthocyanin and PA biosynthesis [22]. Moreover, AtGL3, AtEGL3 and AtMYC1 were shown to regulate trichome formation and root hair patterning [19, 47]. Therefore, it is possible that CsMYC1 and CsbHLH042 may control trichome formation and PA biosynthesis in cucumber.

### Cucumber *bHLH* genes may play important roles in abiotic stress tolerance

In the process of plant response to abiotic stress, bHLH TFs act as regulatory genes to regulate the expression changes of related stress genes, thus playing an important role in stress responses. Many studies have shown that bHLH TFs can respond to a range of stresses. For example, in addition to being involved in the morphogenesis of stomata, the TFs INDUCER OF CBF EXPESSION1 (ICE1) and ICE2 in *Arabidopsis* and their homologous genes in other species can play key roles in the response to low temperature stress [31, 46]. *RERJ1* is upregulated in the event of physical damage and drought stress to plants [48]. All these examples indicate that bHLH TFs can play a certain role in response to abiotic stress. However, little is known about the functions of the *bHLH* gene family in cucumber. To better analyse the protein functions of the *bHLH* gene family in cucumber, we conducted a preliminary analysis of three aspects to reveal the functions of the *CsbHLH* gene family.

How *cis*-elements in the promoters of the *bHLH* genes respond to the environment will affect their roles in stimulating and regulating gene expression. *Cis*-element analyses indicated that there were a wide range of elements on the gene promoters of *CsbHLH* responding to different stresses, such as TCA-element, MBS and LTR (Fig. S3). MYB binding site involved in drought-inducibility existed in many Cs*bHLH* gene promoters (Table S3), indicating that MYB TFs may regulate Cs*bHLHs* expression in drought stress. The TC-rich and ABRE elements related to ABA-dependent or independent stress tolerance also appeared in some Cs*bHLH* gene promoters [49]. In general, according to the *cis*-acting element contained on the promoters, these Cs*bHLH* genes might play key roles responsing to various stresses in cucumber. In addition, the functions of 50 CsbHLHs were predicted, which were mainly related to stress responses and development processes (Table S4). For the third aspect, the regulatory networks for 142 *CsbHLH* genes were predicted, suggesting that a number of genes could respond to stimuli (Table S6). For example, *bHLH093* and *ICE1* were involved in the ABA signalling pathway, which were crucial for abiotic stress responses in plants [49, 50]. These results suggested that the *bHLH* gene family may also be involved in the response to stress, metabolic regulation, and plant development in cucumber, consistent with previous research [10, 12]. Subsequently, we analysed and screened *CsbHLH* genes that might respond to stress, as it is very important to improve stress tolerance of cucumber. According to *cis*-element analyses, the promoter regions of 60 *CsbHLH*s were rich in TC-rich *cis*-elements, suggesting that they may be involved in stress responses and defense (Fig. S3). Moreover, the promoters of 106 *CsbHLHs* contained the ABA-responsive element, responding to ABA stress and 41 *CsbHLHs* contained the LTR element, responding to cold stress. The phylogenetic analyses between *Arabidopsis* and cucumber further showed that 25 *CsbHLHs* might respond to abiotic stresses, such as ABA, salt, cold and drought (Table S4). Through comprehensive analysis, we carefully screened 21, 20 and 25 *bHLH* genes that were likely to respond to low temperature (4 °C), salt (NaCl) and ABA, respectively. The screened *CsbHLH* genes all responded to stress treatments under the respective stress conditions (Fig. 5). *CsbHLH041* was induced by salt and ABA (Fig. 5a-b), and *35S*:*CsbHLH041* transgenic *Arabidopsis thaliana* and transient transformed cucumber cotyledons were shown to have enhanced tolerance to salt and ABA (Fig. 6; Fig. 7). In general, these results provided a good reference for further functional studies of *CsbHLH* gene family in cucumber.

## Conclusions

Our study investigated the *bHLH* family genes in detail in cucumber. We also performed expression analyses of the selected genes under different stress treatments, and detailed functions of *CsbHLH041* using the transgenic method. This work provides new insights into the functions and regulatory mechanisms of CsbHLH proteins in cucumber abiotic stress tolerance and growth and development.

## Methods

### Genome-wide identification of the *CsbHLH* genes in *cucumber*

To identify the *CsbHLH* gene family members from the entire cucumber genome database, 166 *Arabidopsis* bHLH proteins were used as query sequences and BlastP searches against the predicted cucumber proteins. In addition, the Hidden Markov Model (HMM) profile of the bHLH domain (PF00010) from the Pfam database (available online: http://pfam.janelia.org) was also applied as a query to search the *bHLH* genes. We further examined the bHLH domains of all candidate *bHLH* genes as described by [24].

### Phylogenetic analysis and multiple sequence alignment

The sequence logos for bHLHs were obtained by submitting the multiple alignment sequences to the website (http://weblogo.berkeley.edu/logo.cgi) [51]. A phylogenetic tree was constructed with the aligned fully predicted protein sequences of 142 *bHLH* genes using MEGA7 (https://www.megasoftware.net/) [52]. The neighbour-joining (NJ) method was used with the following parameters: Poisson correction, pairwise deletion, and bootstrap (1000 replicates; random seed). The phylogenetic tree was visualized by plotting it using the EvolView tool (http://www.evolgenius.info). Classification of the *CsbHLH* genes was then performed according to their phylogenetic relationships with their corresponding *Arabidopsis bHLH* genes. Multiple sequence alignments were performed as described by [26].

### Conserved motif and gene structure analysis

The 142 *CsbHLH* gene structures were analysed as described by [53]. Conserved motif structures in CsbHLHs were identified using MEME (http://meme-suite.org/index.html) [26].

### Gene duplication and chromosomal distribution

The gene duplication events were assessed as described by [54]. According to the physical location information in the cucumber genome database, 142 *CsbHLH* genes were mapped to cucumber chromosomes as described by [26], and the syntenic analysis maps were completed using TBtools [26].

### Analysis of the *bHLH* gene promoter in cucumber

We downloaded the entire cucumber genome sequence from the cucumber genome database (Chinese Long 9930) and extracted the 2-kb long sequences upstream of the transcription start site of these 142 *CsbHLH* genes. The cis-acting elements on the promoter regions of these genes were analysed using PlantCARE (http://bioinformatics.psb.ugent.be/webtools/plantcare/html/) software [55].

### Plant materials and growth conditions

Cucumber (*Cucumis sativus* L. cv ‘Xintaimici’) seeds, provided by Professor Chenxing Cao (Shandong Agricultural University), were germinated on moist filter paper in an incubator at 28 °C for 1 day. The germinated seeds were sown into soil mixture in an ordinary illuminated incubator at Shandong Agricultural University. After 10 days, batches of 12 seedlings were transferred to a plastic tank filled with an aerated nutrient solution (pH 6.0–6.5) containing the following: Ca (NO_3_)_2_: 3.5 mM, KNO_3_: 7 mM, KH_2_PO_4_: 0.78 mM, MgSO_4_: 2 mM, H_3_BO_3_: 29.6 μM, MnSO_4_: 10 μM, Fe-EDTA: 50 μM, ZnSO_4_: 1.0 μM, H_2_MoO_4_: 0.05 μM and CuSO_4_: 0.95 μM. The experiment was carried out as previously described [56].

### RNA extraction and qRT-PCR analysis

Total RNA was isolated from cucumber and *Arabidopsis* plants using an RNAprep pure Plant Kit (TianGen, Beijing, China), following the manufacturer’s instructions. Subsequently, reverse transcribed using the PrimeScript®1st Strand cDNA Synthesis Kit (Takara, Japan). The qRT-PCR reactions were performed using the UltraSYBR Mixture (with ROX I; Cwbiotech) with the iCycler iQ5 system (BioRad, CA, USA). The results were normalized to those of the cucumber *ACTIN* gene. Three biological replicates were used for each analysis. The primers used in this study are provided in Table S7.

### Overexpression vector construction, *Arabidopsis* transformation and transient transformation in cucumber cotyledons

The full-length coding sequence of *CsbHLH041* was recombined into the pCAMBIA1300 vector. The construct was transformed into *Agrobacterium tumefaciens* LBA4404, which was used for transformation of *Arabidopsis* plants and 8-d-old cucumber cotyledons [57]. The *Arabidopsis* seeds were Colombia (Col-0), which were bred in our laboratory. Homozygous T3 transgenic *Arabidopsis* lines were identified by hygromycin (300 mg/L) selection.

### Abiotic stress tolerance assays and ABA sensitivity analysis

For *Arabidopsis* salt stress and ABA treatment, the seeds of *CsbHLH041* T3-generation homozygous lines and Col-0 (WT) were sown in vermiculite soil in pots and cultured under normal conditions at 22 °C for 3 weeks. For salt treatment, the 3-week-old seedlings were watered with 200 mM NaCl solution every other day, and the growth of Col-0 (WT) and *CsbHLH041* transgenic lines was observed every 4 days. For ABA treatments, the 3-week-old seedlings were watered with 100 μM ABA solution every other day, and phenotypes were evaluated every 4 days. To check the seed germination rate in response to salt stress and ABA treatment, the seeds of Col-0 (WT) and transgenic lines were surface sterilized and sown in 1/2 MS medium supplemented with 2 μM ABA or 100 mM NaCl, respectively, under normal conditions at 22 °C in a growth chamber. The germination rate was scored on the 7th day after culturing on the plates.

To determine the salt tolerance and ABA sensitivity in cotyledons of 8-d-old cucumber seedlings with transient infiltration of *35S* and *35S*:*CsbHLH041*, selected seedlings with equivalent growth were transferred to 6 L nutrient solution for hydroponic growth. Hoagland nutrient solution was used for culture, and the seedlings were grown hydroponically for 2 days before salt and ABA treatment. They were then treated with salt and ABA, and the final concentration in the medium was 100 mM and 100 μM, respectively. To ensure the reliability of the experiment, the cucumber seedlings with transient infiltration of *35S* and *35S*:*CsbHLH041* were cultured in the same hydroponic box. The changes in transgenic and control seedlings were observed at different time periods.

### Determination of physiological parameters

The cucumber cotyledons of *35S* empty vector and *35S:CsbHLH041* seedlings were collected at different time points during salt and ABA stress treatment, then frozen in liquid nitrogen for subsequent experiments. The activity of superoxide dismutase (SOD), peroxidase (POD), and catalase (CAT) were determined as previously described [58].

### The functional annotations and protein association network predictions

We submitted the 142 CsbHLH protein sequences to the online server (version 10.0; http://string-db.org). For details was as described by [40].

## Supplementary information

**Additional file 1.** Supplementary Figs. S1 to S4. (Fig. S1. Ten conserved motifs from 142 CsbHLH proteins; Fig. S2. Genome locations of the 142 *CsbHLH* genes on 7 chromosomes; Fig. S3. Cis-element analysis in the *CsbHLH* genes promoter regions; Fig. S4. Co-expression network of the *CsbHLH* genes).

**Additional file 2: Table S1.** Tandem duplication and Segmental duplication events.

**Additional file 3: Table S2.** Synteny analysis of *bHLH* genes in cucumber, *Arabidpsis* and tomato.

**Additional file 4: Table S3.***Cis*-elements in the promoters of 142 *CsbHLH* genes.

**Additional file 5: Table S4.** Predicted functions of *CsbHLHs* with the function of their homologs verified in *Arabidopsis* by phylogenetic analysis.

**Additional file 6: Table S5.** Syntenic blocks between the cucumber and *Arabidopsis* and tomato genome.

**Additional file 7: Table S6.** String protein annotations.

**Additional file 8: Table S7.** Primers used for qRT-PCR.

**Additional file 9.** Gel image.

## Data Availability

The data that support the results are included within the article and its additional files. Other relevant materials are available from the corresponding authors on reasonable request.

## Abbreviations

bHLH: Basic Helix-Loop-Helix
At: *Arabidopsis thaliana*
Cs: *Cucumis sativus* L
MS: Murashige and Skoog
qRT-PCR: Quantitative reverse transcription-PCR
CDS: Coding Sequence
ABA: Abscisic acid
pI: Isoelectric point
WT: Wild type
