# Supplementary material for: Genome-wide identification and characterization of cucumber bHLH family genes and the functional characterization of CsbHLH041 in NaCl and ABA tolerance in Arabidopsis and cucumber
Source: BMC Plant Biol. 2020 Jun 11;20:272. doi: 10.1186/s12870-020-02440-1 (PMC7291561; doi:10.1186/s12870-020-02440-1)
Supplement: Supplementary file 1 — Additional file 1. Supplementary Figs. S1 to S4. (Fig. S1. Ten conserved motifs from 142 CsbHLH proteins; Fig. S2. Genome locations of the 142 CsbHLH genes on 7 chromosomes; Fig. S3. Cis-element analysis in the CsbHLH genes promoter regions; Fig. S4. Co-expression network of the CsbHLH genes). [file 12870_2020_2440_MOESM1_ESM.pdf]

Logo	E-value	Sites	Width
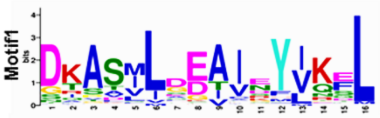	3.0e-813	130	16
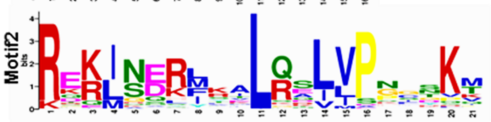	1.9e-941	124	21
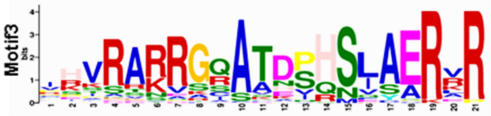	5.8e-376	48	21
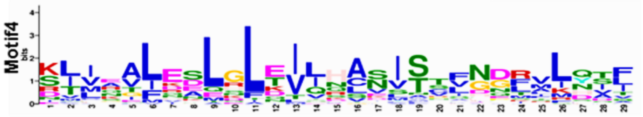	2.3e-148	39	29
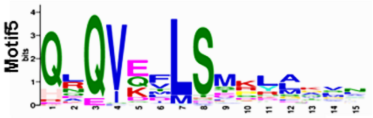	7.7e-134	60	15
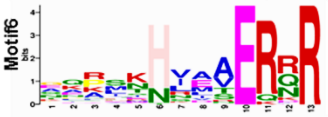	1.5e-107	48	13
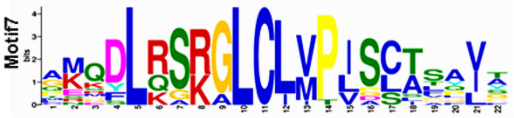	9.3e-086	15	22
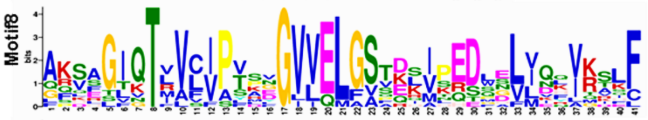	2.4e-091	13	41
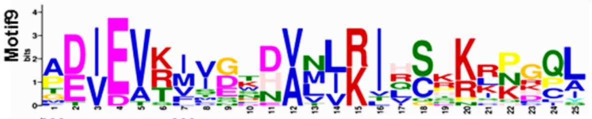	1.3e-072	16	25
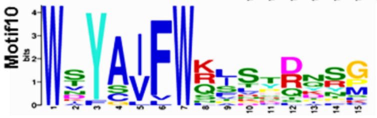	3.3e-065	17	15

Fig. S1. Ten conserved motifs from 142 CsbHLH proteins. The logos of ten conserved motif sequences were obtained from MEME Suite website. The bit score means information content of each position in the amino acid sequence.

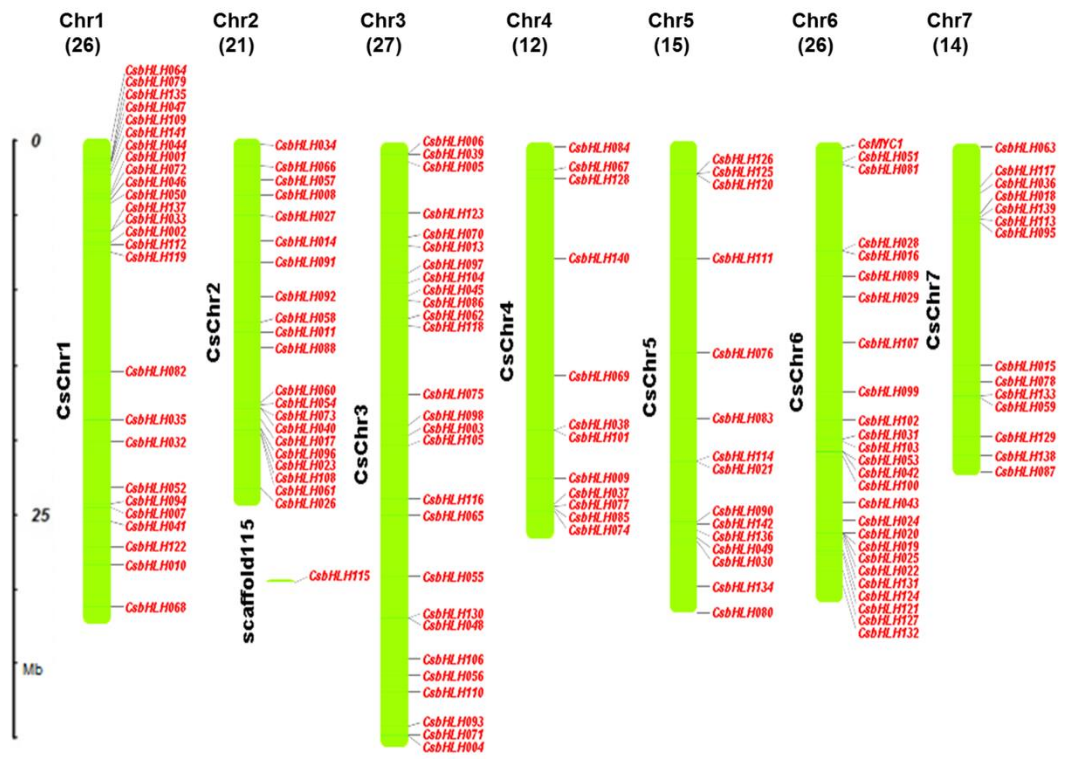

Fig. S2. Genome locations of the 142 *CsbHLH* genes on 7 chromosomes. Chromosomal positions of the *CsbHLH* genes were indicated by *CsbHLH* number. The scale was in megabases (Mb). The numbers below the name of the chromosome showed the number of CsbHLH genes in this chromosome.

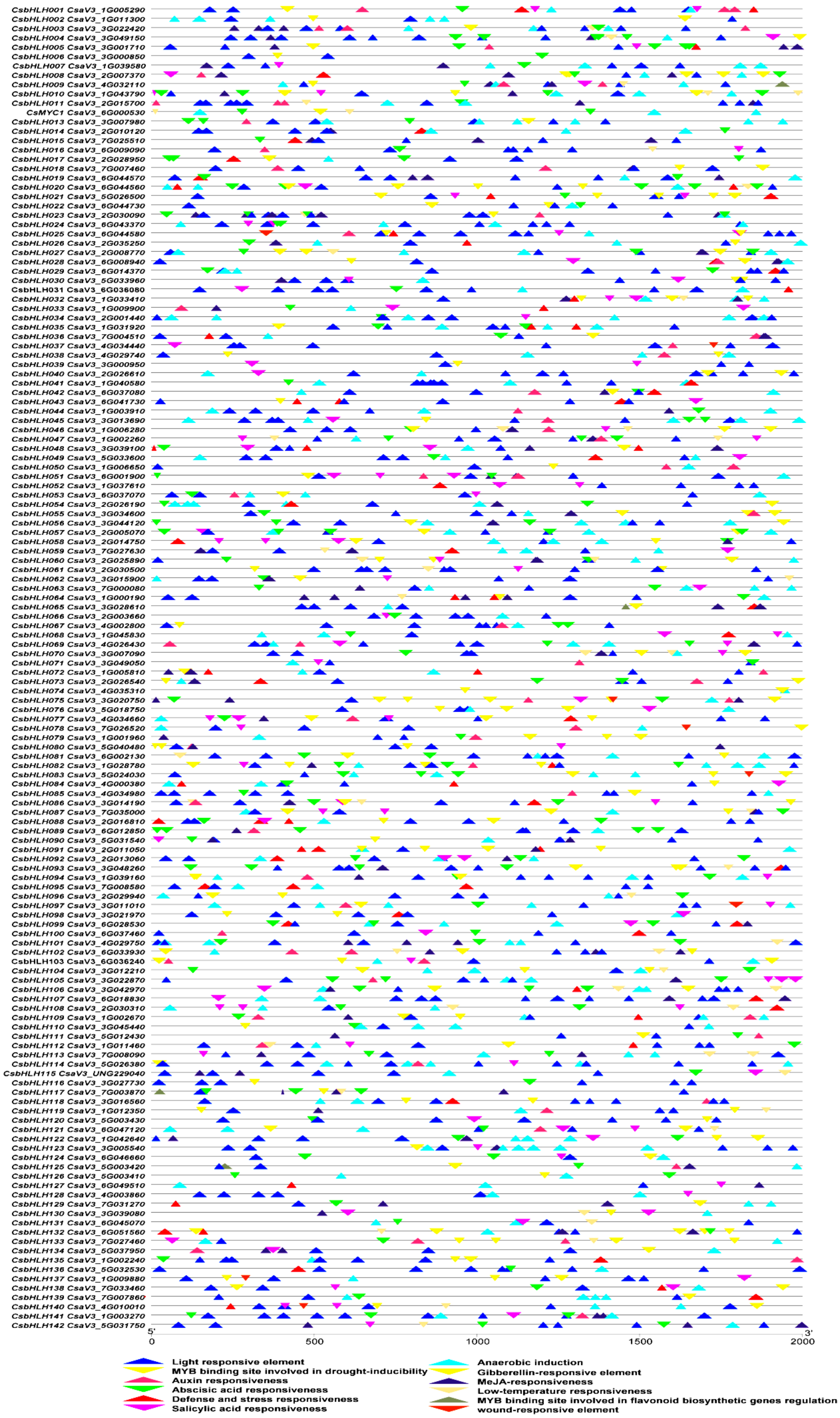
CsbHLH001 CsaV3_1G005290
CsbHLH002 CsaV3_1G011300
CsbHLH003 CsaV3_3G022420
CsbHLH004 CsaV3_3G049150
CsbHLH005 CsaV3_3G001710
CsbHLH006 CsaV3_3G000850
CsbHLH007 CsaV3_1G039580
CsbHLH008 CsaV3_2G007370
CsbHLH009 CsaV3_4G032110
CsbHLH010 CsaV3_1G043790
CsbHLH011 CsaV3_2G015700
CsMYC1 CsaV3_6G000530
CsbHLH013 CsaV3_3G007980
CsbHLH014 CsaV3_2G010120
CsbHLH015 CsaV3_7G025510
CsbHLH016 CsaV3_6G009090
CsbHLH017 CsaV3_2G028950
CsbHLH018 CsaV3_7G007460
CsbHLH019 CsaV3_6G044570
CsbHLH020 CsaV3_6G044560
CsbHLH021 CsaV3_5G026500
CsbHLH022 CsaV3_6G044730
CsbHLH023 CsaV3_2G030090
CsbHLH024 CsaV3_6G043370
CsbHLH025 CsaV3_6G044580
CsbHLH026 CsaV3_2G035250
CsbHLH027 CsaV3_2G008770
CsbHLH028 CsaV3_6G008940
CsbHLH029 CsaV3_6G014370
CsbHLH030 CsaV3_5G033960
CsbHLH031 CsaV3_6G036080
CsbHLH032 CsaV3_1G033410
CsbHLH033 CsaV3_1G009900
CsbHLH034 CsaV3_2G001440
CsbHLH035 CsaV3_1G031920
CsbHLH036 CsaV3_7G004510
CsbHLH037 CsaV3_4G034440
CsbHLH038 CsaV3_4G029740
CsbHLH039 CsaV3_3G000950
CsbHLH040 CsaV3_2G026610
CsbHLH041 CsaV3_1G040580
CsbHLH042 CsaV3_6G037080
CsbHLH043 CsaV3_6G041730
CsbHLH044 CsaV3_1G003910
CsbHLH045 CsaV3_3G013690
CsbHLH046 CsaV3_1G006280
CsbHLH047 CsaV3_1G002260
CsbHLH048 CsaV3_3G039100
CsbHLH049 CsaV3_5G033600
CsbHLH050 CsaV3_1G006650
CsbHLH051 CsaV3_6G001900
CsbHLH052 CsaV3_1G037610
CsbHLH053 CsaV3_6G037070
CsbHLH054 CsaV3_2G026190
CsbHLH055 CsaV3_3G034600
CsbHLH056 CsaV3_3G044120
CsbHLH057 CsaV3_2G005070
CsbHLH058 CsaV3_2G014750
CsbHLH059 CsaV3_7G027630
CsbHLH060 CsaV3_2G025890
CsbHLH061 CsaV3_2G030500
CsbHLH062 CsaV3_3G015900
CsbHLH063 CsaV3_7G000080
CsbHLH064 CsaV3_1G000190
CsbHLH065 CsaV3_3G028610
CsbHLH066 CsaV3_2G003660
CsbHLH067 CsaV3_4G002800
CsbHLH068 CsaV3_1G045830
CsbHLH069 CsaV3_4G026430
CsbHLH070 CsaV3_3G007090
CsbHLH071 CsaV3_3G049050
CsbHLH072 CsaV3_1G005810
CsbHLH073 CsaV3_2G026540
CsbHLH074 CsaV3_4G035310
CsbHLH075 CsaV3_3G020750
CsbHLH076 CsaV3_5G018750
CsbHLH077 CsaV3_4G034660
CsbHLH078 CsaV3_7G026520
CsbHLH079 CsaV3_1G001960
CsbHLH080 CsaV3_5G040480
CsbHLH081 CsaV3_6G002130
CsbHLH082 CsaV3_1G028780
CsbHLH083 CsaV3_5G024030
CsbHLH084 CsaV3_4G000380
CsbHLH085 CsaV3_4G034980
CsbHLH086 CsaV3_3G014190
CsbHLH087 CsaV3_7G035000
CsbHLH088 CsaV3_2G016810
CsbHLH089 CsaV3_6G012850
CsbHLH090 CsaV3_5G031540
CsbHLH091 CsaV3_2G011050
CsbHLH092 CsaV3_2G013060
CsbHLH093 CsaV3_3G048260
CsbHLH094 CsaV3_1G039160
CsbHLH095 CsaV3_7G008580
CsbHLH096 CsaV3_2G029940
CsbHLH097 CsaV3_3G011010
CsbHLH098 CsaV3_3G021970
CsbHLH099 CsaV3_6G028530
CsbHLH100 CsaV3_6G037460
CsbHLH101 CsaV3_4G029750
CsbHLH102 CsaV3_6G033930
CsbHLH103 CsaV3_6G036240
CsbHLH104 CsaV3_3G012210
CsbHLH105 CsaV3_3G022870
CsbHLH106 CsaV3_3G042970
CsbHLH107 CsaV3_6G018830
CsbHLH108 CsaV3_2G030310
CsbHLH109 CsaV3_1G002670
CsbHLH110 CsaV3_3G045440
CsbHLH111 CsaV3_5G012430
CsbHLH112 CsaV3_1G011460
CsbHLH113 CsaV3_7G008090
CsbHLH114 CsaV3_5G026380
CsbHLH115 CsaV3_UNG229040
CsbHLH116 CsaV3_3G027730
CsbHLH117 CsaV3_7G003870
CsbHLH118 CsaV3_3G016560
CsbHLH119 CsaV3_1G012350
CsbHLH120 CsaV3_5G003430
CsbHLH121 CsaV3_6G047120
CsbHLH122 CsaV3_1G042640
CsbHLH123 CsaV3_3G005540
CsbHLH124 CsaV3_6G046660
CsbHLH125 CsaV3_5G003420
CsbHLH126 CsaV3_5G003410
CsbHLH127 CsaV3_6G049510
CsbHLH128 CsaV3_4G003860
CsbHLH129 CsaV3_7G031270
CsbHLH130 CsaV3_3G039080
CsbHLH131 CsaV3_6G045070
CsbHLH132 CsaV3_6G051560
CsbHLH133 CsaV3_7G027460
CsbHLH134 CsaV3_5G037950
CsbHLH135 CsaV3_1G002240
CsbHLH136 CsaV3_5G032530
CsbHLH137 CsaV3_1G009880
CsbHLH138 CsaV3_7G033460
CsbHLH139 CsaV3_7G007860
CsbHLH140 CsaV3_4G010010
CsbHLH141 CsaV3_1G003270
CsbHLH142 CsaV3_5G031750
5'
3'
0
500
1000
1500
2000
Light responsive element
MYB binding site involved in drought-inducibility
Auxin responsiveness
Abscisic acid responsiveness
Defense and stress responsiveness
Salicylic acid responsiveness
Anaerobic induction
Gibberellin-responsive element
MeJA-responsiveness
Low-temperature responsiveness
MYB binding site involved in flavonoid biosynthetic genes regulation
wound-responsive element

Fig. S3. *Cis*-element analysis in the *CsbHLH* genes promoter regions. The potential *cis*-regulatory elements in the promoter regions 2-kb upstream of the *CsbHLH* genes, particularly the elements related to stress responsiveness (light induction; anaerobic induction, low temperature and drought inducibility) and plant hormones (Abscisic acid, Auxin, MeJA, Gibberellic acid and Salicylic acid). Different colored triangles represented different *cis*-elements.

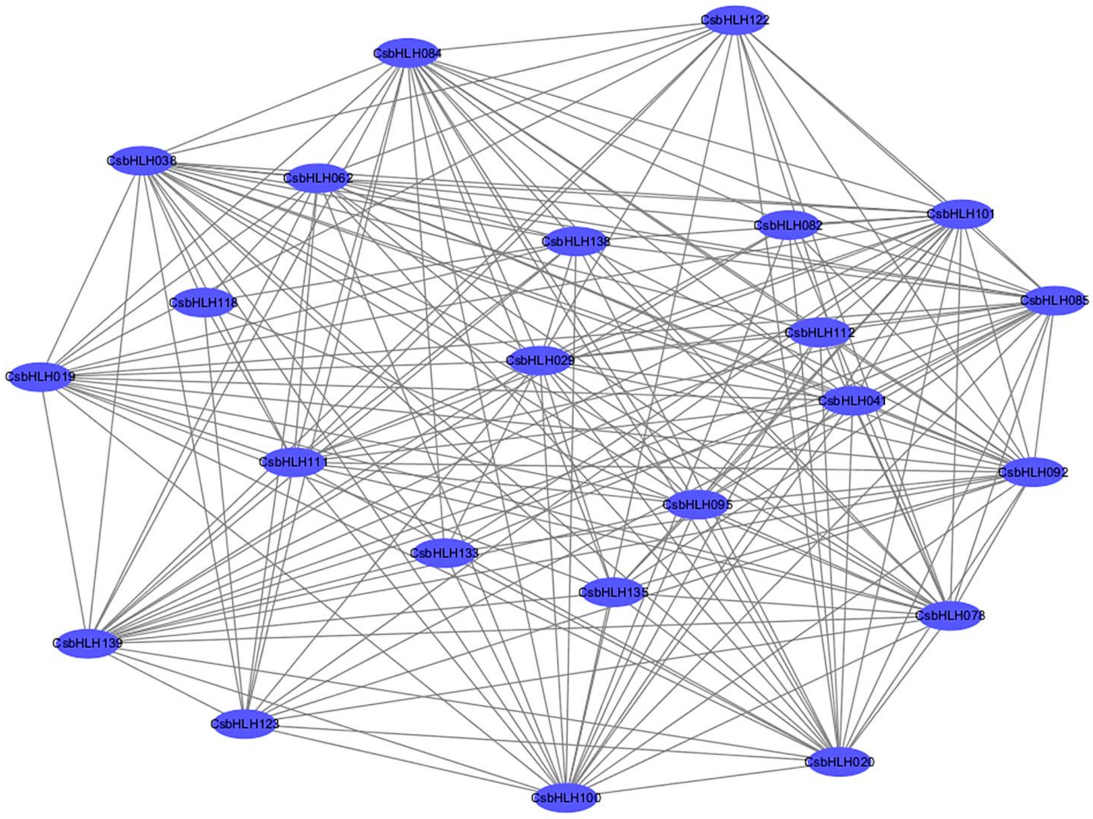

Fig. S4. Co-expression network of the *CsbHLH* genes. Using the available RNA-seq data of 10 tissues regarding correlations between cucumber *bHLH* genes, a network of co-expressed genes with weight value greater than 0.5 was constructed This network contains 23 *CsbHLH* genes (nodes) and 191 correlations (edges).
