# Supplementary figures and images for: Genome-wide identification and characterization of cucumber bHLH family genes and the functional characterization of CsbHLH041 in NaCl and ABA tolerance in Arabidopsis and cucumber
Source: BMC Plant Biol. 2020 Jun 11;20:272. doi: 10.1186/s12870-020-02440-1 (PMC7291561; doi:10.1186/s12870-020-02440-1)

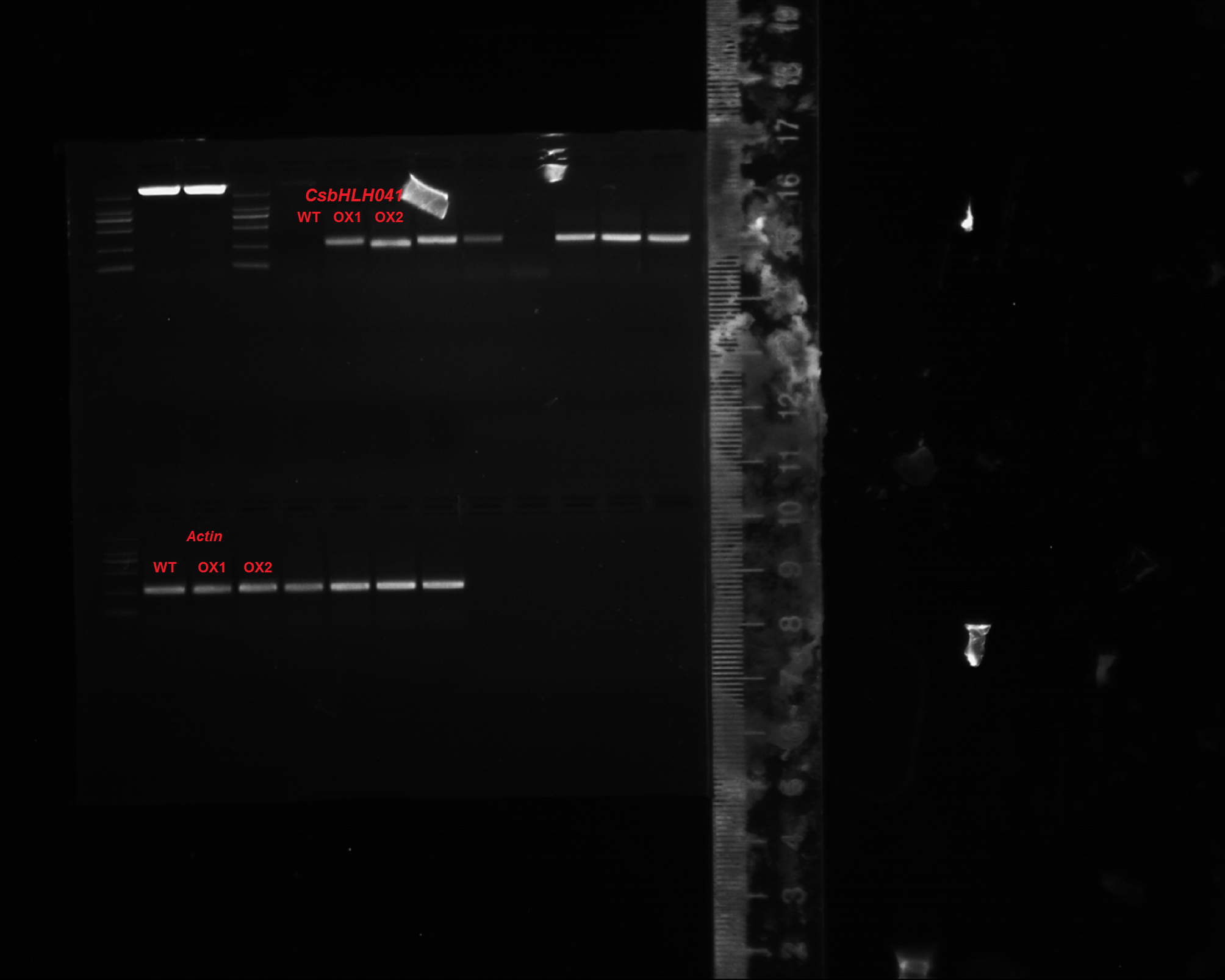

Supplement: Supplementary file 9 — Additional file 9. Gel image. [file 12870_2020_2440_MOESM9_ESM.tif]
